# Genomic Instability Score Across Diverse Tumor Types Using the Illumina TruSight Oncology 500 HRD Assay

**DOI:** 10.3390/diagnostics16121802

**Published:** 2026-06-11

**Authors:** Moonsik Kim, An Na Seo, Nora Jee-Young Park, Ghilsuk Yoon, Ji Young Park

**Affiliations:** Department of Pathology, School of Medicine, Kyungpook National University, Kyungpook National University Chilgok Hospital, Daegu 41404, Republic of Korea; teiroa83@knu.ac.kr (M.K.);

**Keywords:** genomic instability score, homologous recombination deficiency, TruSight Oncology 500 HRD, next-generation sequencing, *BRCA1/2*

## Abstract

**Background:** Homologous recombination deficiency (HRD) is emerging as a clinically relevant biomarker across diverse tumor types, in addition to ovarian cancer. In this study, we evaluated the genomic instability score (GIS) across multiple tumor types using the TruSight Oncology 500 HRD assay, which incorporates the Myriad Genetics GIS algorithm, a widely used reference standard for HRD assessment. **Methods:** A total of 162 tumor samples (17 ovarian cancers and 145 non-ovarian tumors) underwent next-generation sequencing using the TruSight Oncology 500 HRD assay. **Results:** A total of 14 tumors were classified as GIS-High, defined as a GIS score ≥42, representing 8.6% of all cases. Among ovarian cancers, 7 out of 17 cases (41.2%) met the GIS-High threshold. Among non-ovarian tumors, seven GIS-High tumors were identified, accounting for 4.8% of cases (7/145). GIS-High cases occurred in breast (*n* = 4), lung (*n* = 2), and hepatobiliary tract (*n* = 1) cancers. GIS scores showed significant associations with *BRCA1/2* and *TP53* mutational status. In contrast, alterations in HRD-related genes other than *BRCA1/2* did not show significant associations with GIS score. **Conclusions:** GIS-High tumors were identified in a small subset of non-ovarian cancers. These findings support further investigation of GIS as an exploratory biomarker of HRD-like genomic scarring beyond ovarian cancer, but its predictive and therapeutic relevance in non-ovarian tumors requires additional validation.

## 1. Introduction

Next-generation sequencing (NGS) has been widely adopted to identify actionable molecular alterations in the era of precision medicine [1]. Unlike conventional molecular techniques such as Sanger sequencing and polymerase chain reaction, which typically interrogate a limited number of genetic alterations per assay, NGS enables the simultaneous detection of numerous variants, including single-nucleotide variants, small insertions/deletions, gene fusions, and splice variants [2]. Beyond identifying genetic alterations and structural variations, NGS assays can also provide several clinically relevant biomarkers, including tumor mutational burden (TMB) [3], mutational signatures [4], microsatellite instability (MSI) status [5], and homologous recombination deficiency (HRD) status, a recently emerging biomarker [6]. Complementary computational approaches, such as signaling entropy-based pathway activation analysis, may also provide pathway-level interpretations of cancer biology [7]. In addition, researchers have begun integrating microbiome, transcriptomic, and histopathologic data through emerging multi-omics approaches to improve cancer risk stratification [8].

DNA double-strand breaks are repaired primarily through either the homologous recombination (HR) pathway or non-homologous end joining (NHEJ). The HR pathway uses an intact sister chromatid as a repair template and is therefore less error-prone than NHEJ [9]. Key genes involved in the HR pathway include *BRCA1*, *BRCA2*, *PALB2*, *ATM*, *ATR*, and others. Malignant tumors can acquire HRD through diverse genetic and epigenetic alterations [10]. HRD-positive tumors are selectively vulnerable to poly (ADP-ribose) polymerase (PARP) inhibitors via the mechanism of “synthetic lethality” [11,12]. Although HRD is most frequently observed in ovarian cancer, where tumorigenesis is closely linked to defects in the HR pathway, HRD has also been reported in other tumor types, including breast and prostate cancers [10].

Although many commercial and research-use NGS panels now report HRD status, the FDA-approved Myriad myChoice CDx assay, which integrates loss of heterozygosity (LOH) [13], telomeric allelic imbalance (TAI) [14], and large-scale state transitions (LST) [15] into a genomic instability score (GIS; with HRD-positivity defined as a score ≥42), is currently regarded as the gold standard for HRD assessment [10,16]. Several previous studies have explored HRD across multiple tumor types. However, approaches based solely on alterations in HRD-related genes (i.e., panel-based HRD detection) have limitations in that they do not directly capture the downstream genomic scars of HRD, such as LOH [17]. Although some studies have quantified LOH across diverse tumor types, they often did not incorporate other characteristic genomic-scar features of HRD, including LST and TAI [18]. The Illumina TruSight Oncology 500 HRD (TSO500 HRD) assay is a targeted NGS panel that applies the Myriad Genetics GIS algorithm to generate the GIS score, and validation studies have shown excellent concordance between the GIS score generated by the TSO500 HRD assay and those obtained with the Myriad myChoice CDx test.

In this study, we investigated GIS across diverse tumor types using the TSO500 HRD assay and evaluated its associations with driver alterations and other clinically relevant biomarkers.

## 2. Materials and Methods

### 2.1. Study Samples

We retrospectively collected 162 consecutive tumor samples that had undergone clinician-ordered TSO500 HRD testing at Kyungpook National University Chilgok Hospital from August to December 2025. All specimens included in this study were patient-derived clinical samples submitted for clinician-ordered TSO500 HRD testing, rather than research-only specimens. Clinicians ordered TSO500 HDR testing as part of routine practice to support diagnostic evaluation and therapeutic decision-making. The cohort included 91 biopsy samples, 66 resection samples, and 5 cytology samples. We retrieved clinicopathologic data from the hospital’s electronic medical records including patient age, sex, histologic subtype, sampling method, programmed death-ligand 1 (PD-L1) score, and NGS results. PD-L1 immunohistochemistry was performed in 68 cases (42.0%) based on clinical indication. Cases not tested for PD-L1 were recorded separately and were not considered PD-L1 negative. For lung cancers, PD-L1 positivity was defined as a tumor proportion score (TPS) ≥1%. For non-lung tumors in which PD-L1 testing was clinically indicated, PD-L1 positivity was defined as a combined positive score (CPS) ≥1, according to tumor-specific clinical practice and assay interpretation criteria [19,20]. Because scoring methods varied across tumor types, we incorporated PD-L1 status into the biomarker distribution analysis only as a binary (positive/negative) variable, based on clinically used tumor-specific scoring criteria.

### 2.2. Next-Generation Sequencing

After histopathologic review, only tumor samples with tumor cellularity greater than 20% were submitted for sequencing. Five-micrometer-thick sections were cut from formalin-fixed, paraffin-embedded (FFPE) tissue blocks, deparaffinized in xylene, and rehydrated through graded ethanol solutions. DNA and RNA were extracted using the AllPrep DNA/RNA Kit (Qiagen Sciences, Germantown, MD, USA). The minimum nucleic acid input requirements were 120 ng of DNA in 60 μL and 80 ng of RNA in 8.5 μL. Samples that did not meet these pre-analytical requirements were not submitted for sequencing. Library preparation and target enrichment were performed using the TruSight Oncology 500 HRD (TSO500 HRD) Kit (Illumina, San Diego, CA, USA), and sequencing was performed on the NextSeq 550 platform (Illumina). Supplementary Table S1 summarizes the key sequencing QC metrics, including run-level Q30 metrics, median exon coverage, percentage of exon targets covered at ≥50×, HRD target coverage, MSI-usable sites, and CNV-related QC metrics. The DRAGEN TruSight Oncology 500 pipeline (version 2.6.0) was used for data processing, alignment, variant calling, and biomarker assessment, including TMB, MSI, and GIS. Variant annotation and interpretation were conducted with Illumina Connected Insights (version 5.2.0). GIS-High was defined as GIS score ≥42; TMB-High as ≥10.0 mutations/Mb; MSI-High as ≥15.0% MSI unstable sites. Somatic variants were interpreted using the AMP/ASCO/CAP guidelines for somatic variant interpretation. “Targetable genetic alterations” were defined as Tier I variants, meaning that they have strong clinical significance based on FDA-approved therapies, professional guidelines, or well-established clinical evidence [21]. All variants were interpreted in a tumor-type-specific context. Other than *BRCA1/2*, HRD-related genes included *PALB2*, *ATM*, *ATR*, *CHEK1*, *CHEK2*, *BARD1*, *BRIP1*, *RAD50*, *RAD51B*, *RAD51C*, *RAD51D*, *RAD54L*, *CDK12*, *FANCA*, *FANCC*, *FANCD2*, *FANCE*, *FANCL*, *MRE11*, and *NBN*, as reported in the literature [22,23].

### 2.3. Statistical Analysis

Correlations between continuous variables were assessed using Spearman’s rank correlation coefficient, which was also interpreted as the effect-size estimate for correlation analyses. The Wilcoxon rank-sum test was used for non-parametric comparisons between two independent groups. For group comparisons, GIS scores were summarized as median and interquartile range (IQR), and effect sizes for the Wilcoxon rank-sum tests were calculated where appropriate. The frequency of GIS-High tumors was reported with exact binomial 95% confidence intervals. Specimen type was categorized as resection versus biopsy/cytology for additional analyses assessing potential sampling-related effects on GIS score. To account for multiple exploratory biomarker comparisons, Benjamini–Hochberg false discovery rate adjustment was applied. Multivariable linear regression was performed to assess the association between GIS score and *BRCA1/2* alteration, *TP53* mutation, tumor group, specimen type, age, and sex. Tumor groups were categorized as ovary, breast, lung, hepatobiliary, uterus, and others. Sensitivity analyses were performed after excluding ovarian cancers to evaluate whether the observed biomarker associations were driven primarily by ovarian cancer cases. All *p* values were two-sided, and *p* < 0.05 was considered statistically significant. Statistical analyses were performed using R software (version 4.3.2; R Foundation for Statistical Computing, Vienna, Austria).

## 3. Results

### 3.1. Cohort Characteristics

The cohort characteristics are summarized in Table 1 and Supplementary Figure S1. Among the 162 patient samples, 69 were from male patients and 93 from female patients. The mean patient age was 62.5 years (range, 0–84 years). Targetable genetic alterations (consistent with Tier I criteria), including TMB-High status, were identified in 62.3% of cases (101/162). When TMB-High was excluded, we detected targetable genetic alterations in 50.6% of cases (82/162). We identified MSI-High tumors in 9 cases (5.6%), all originating from the digestive tract or female genital tract. Among the cases tested for PD-L1 positivity, 45 cases (66.2%) were positive. Oncogenic *BRCA1/2* alterations were identified in 8 cases (4.9%), and oncogenic alterations in other HRD-related genes were detected in 7 cases (4.3%). Supplementary Table S2 provides detailed clinicopathologic characteristics and genetic findings.

### 3.2. Sequencing Quality Control

All 162 cases completed every analysis step, and the metrics output showed no failed or skipped steps. The median exon coverage was 505. The median percentage of exon targets covered at ≥50× was 99.2%, the median percentage of HRD targets covered at ≥50× was 98.5%, and the median HRD target coverage was 183.5.

### 3.3. GIS Score Distribution and GIS-High Tumors

**Figure 1:**
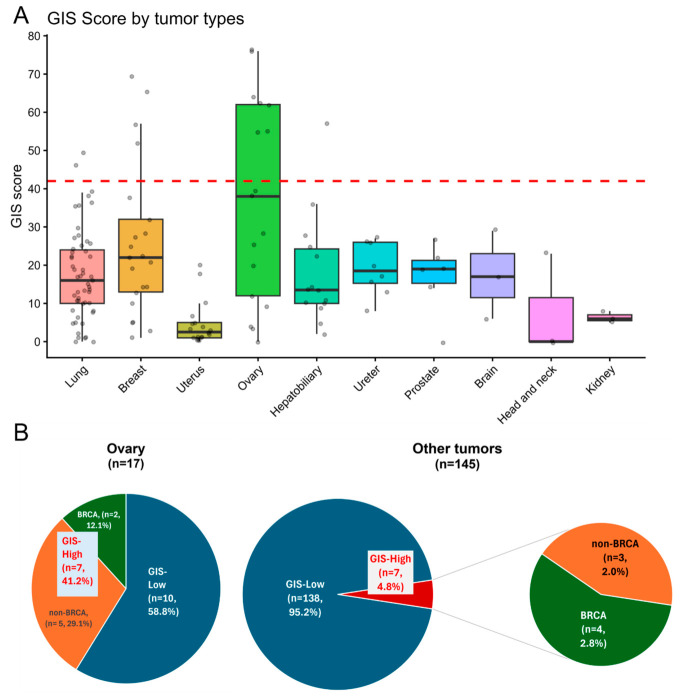
(**A**) Boxplot illustrating the distribution of genomic instability score (GIS) across tumor types. The horizontal red dashed line indicates the predefined cutoff for the GIS-high category (GIS ≥ 42). (**B**) Pie chart showing the proportions of GIS-High tumors by tumor type (ovarian vs. non-ovarian). GIS-High tumors are further stratified by *BRCA1/2* mutational status.

Figure 1 shows the distribution of GIS scores. GIS-High tumors were identified in 14 out of 162 cases (8.6%; exact 95% CI, 4.8–14.1%). GIS scores did not differ significantly by specimen type. Although the biopsy/cytology specimens showed a numerically higher median GIS score than resection specimens (17 [IQR, 10–26] vs. 10.5 [IQR, 4.25–24]), the difference was not statistically significant (*p* = 0.085).

### 3.4. GIS Score in Ovarian Cancer

**Figure 2:**
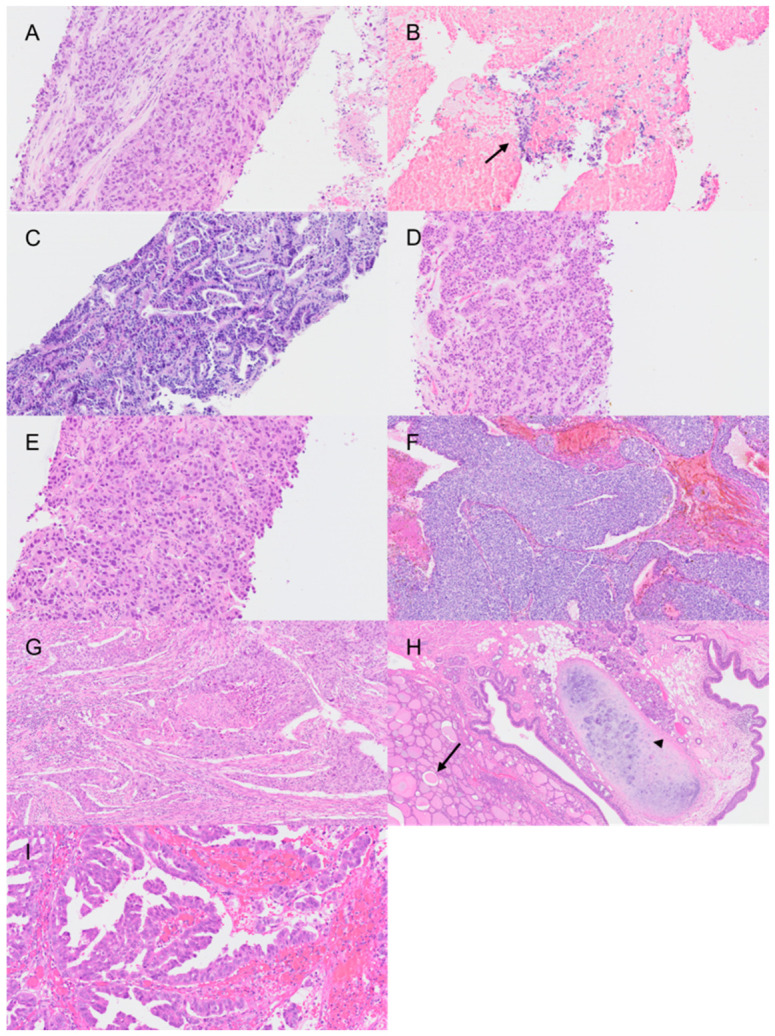
Representative histopathologic images of GIS-High tumors. (**A**,**B**) Lung adenocarcinomas showing poorly differentiated histology. In panel (**B**), abundant fibrinous clot is present, and poorly differentiated adenocarcinoma cells are located mainly in the central portion of the image, as indicated by arrows. Positivity for TTF-1 and absence of neuroendocrine marker expression support the diagnosis of lung adenocarcinoma. (**C**) Hepatobiliary adenocarcinoma of the gallbladder. (**D**–**F**) Invasive ductal carcinoma of the breast, representing luminal, HER2-amplified, and triple-negative subtypes, respectively. (**G**) Ovarian squamous cell carcinoma arising from a mature cystic teratoma. (**H**) Mature cystic teratoma component with chondroid tissue (arrowheads) and thyroid tissue (arrows). (**I**) Ovarian high-grade serous carcinoma with papillary/micropapillary architecture. Original magnifications: (**A**–**E**,**I**) ×200; (**F**,**G**) ×100; (**H)** ×50.

Among the ovarian cancers, 7 out of 17 cases were GIS-High (41.2%; exact 95% CI, 18.4–67.1%) (Table 2). The ovarian cancer cohort included nine high-grade serous carcinomas, three low-grade serous carcinomas, three clear cell carcinomas, one carcinosarcoma, and one squamous cell carcinoma. Of the seven GIS-High ovarian cancers, two harbored oncogenic *BRCA1* alterations, and six (85.7%) were high-grade serous carcinomas. The remaining GIS-High ovarian cancer was a squamous cell carcinoma arising from a mature cystic teratoma (Figure 2).

### 3.5. GIS Score Across Diverse Tumor Types

Figure 1 shows the GIS scores across other tumor types. Seven out of 145 non-ovarian tumors were GIS-High (4.8%; exact 95% CI, 2.0–9.7%), four of which harbored oncogenic *BRCA2* alterations. Four cases were invasive ductal carcinoma of the breast. Interestingly, only one GIS-High breast cancer was triple-negative breast cancer, whereas two were luminal-type tumors and one was HER2-amplified. Two lung adenocarcinomas and one hepatobiliary adenocarcinoma (gallbladder cancer) were classified as GIS-High (Figure 2).

### 3.6. Association Between GIS with BRCA1/2 Alterations, Non-BRCA HRD-Related Gene Alterations, and Other Genomic Biomarkers

**Figure 3:**
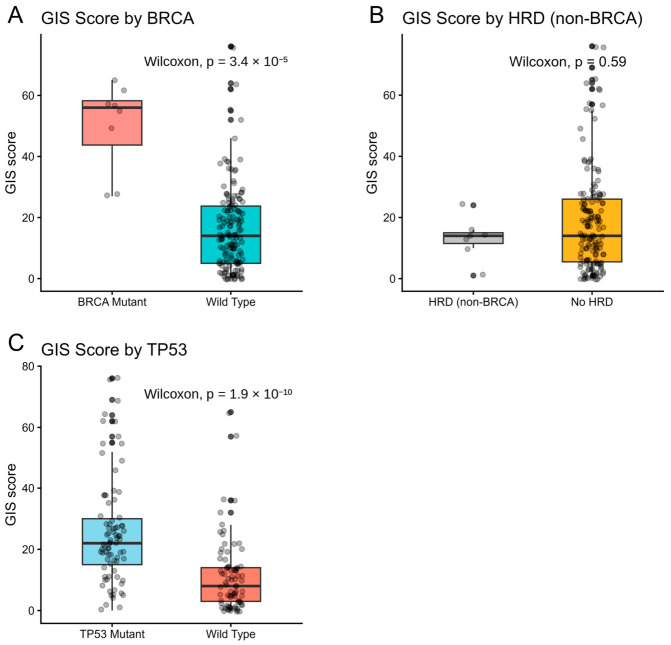
Boxplot illustrating GIS scores according to accompanying genetic alterations, including *BRCA1/2* (**A**), non-*BRCA* homologous recombination–related genes (**B**), and *TP53* (**C**).

We next examined the association between GIS score and other genetic alterations. Tumors harboring *BRCA1/2* alterations had significantly higher GIS scores than *BRCA1/2*-wild-type tumors (median, 56 [IQR, 43.8–58.2] vs. 14 [IQR, 5.0–23.8]; Wilcoxon rank-sum test, raw *p* = 3.38 × 10^−5^; adjusted *p* = 5.07 × 10^−5^; effect size *r* = 0.326; Figure 3). Similarly, *TP53*-mutated tumors had significantly higher GIS scores than *TP53*-wild-type tumors (median, 22 [IQR, 15–30] vs. 8 [IQR, 3–14]; raw *p* = 1.91 × 10^−10^; adjusted *p* = 5.72 × 10^−10^; effect size *r* = 0.501). In sensitivity analyses excluding ovarian cancers, the associations between GIS score and *BRCA1/2* alterations or *TP53* mutation remained significant. Among the non-ovarian tumors, *BRCA1/2*-altered tumors showed higher GIS scores than *BRCA1/2*-wild-type tumors (median, 53 [IQR, 33.2–57] vs. 13 [IQR, 5–22]; adjusted *p* = 0.000264), and *TP53*-mutated tumors also showed higher GIS scores than *TP53*-wild-type tumors (median, 20 [IQR, 11–27] vs. 9 [IQR, 2.75–14]; adjusted *p* = 5.53 × 10^−7^). In multivariable linear regression adjusted for age, sex, sampling method, and tumor group, *BRCA1/2* alteration and *TP53* mutation remained independently associated with higher GIS scores. In sensitivity analyses excluding ovarian cancers, the associations between GIS score and *BRCA1/2* alteration or *TP53* mutation remained significant, whereas alterations in non-*BRCA* HRD-related genes remained unassociated with GIS score.

In contrast, alterations in non-*BRCA* HRD-related genes were not associated with higher GIS scores (median, 14 [IQR, 11.5–15] vs. 14 [IQR, 5.5–26]; raw *p* = 0.595; adjusted *p* = 0.595; effect size *r* = 0.042). This finding remained unchanged after excluding ovarian cancers (adjusted *p* = 0.740).

We also evaluated the relationship between GIS score and TMB and MSI. GIS score showed a weak positive correlation with TMB score (Spearman’s rho = 0.231, raw *p* = 0.003; Benjamini–Hochberg adjusted *p* = 0.006). In contrast, GIS score was not correlated with MSI score (Spearman’s rho = −0.030, raw *p* = 0.704; adjusted *p* = 0.704). Similar findings were observed in the sensitivity analysis excluding ovarian cancers, with a weak positive correlation between the GIS and TMB scores (rho = 0.237, adjusted *p* = 0.008) and no correlation between the GIS and MSI scores (rho = −0.051, adjusted *p* = 0.539) (Supplementary Table S3).

## 4. Discussion

**Figure 4:**
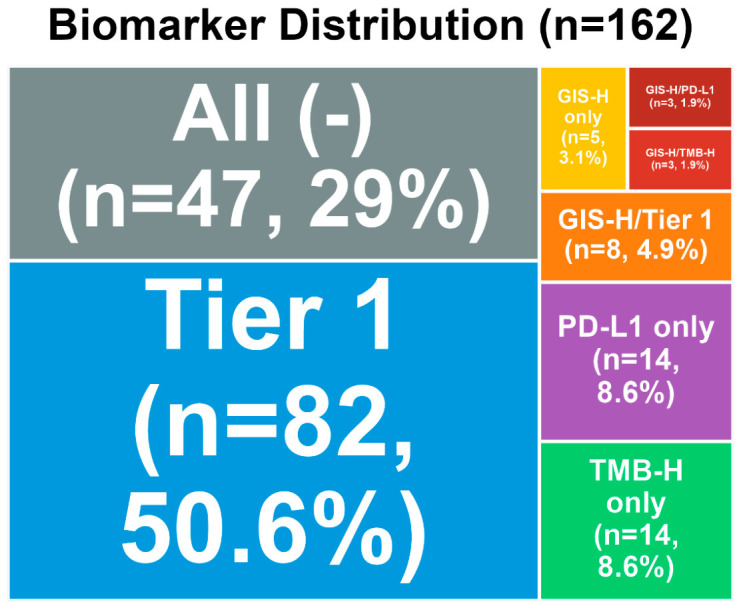
Mosaic plot illustrating the distribution of clinically relevant biomarkers and actionable genetic alterations across all tumor types. Clinically actionable genetic alterations were defined as Tier I alterations according to the AMP/ASCO/CAP guidelines and interpreted in a tumor-type-specific context. GIS-High status alone, without accompanying Tier I alterations, TMB-High status, MSI-High status, or PD-L1 positivity, was observed in 3.1% of tumors.

In this study, we characterized the distribution of GIS scores and examined their associations with clinicopathologic parameters across diverse tumor types. Notably, a subset of tumors met the GIS-High threshold despite lacking Tier I alterations, TMB-High status, MSI-High status, or PD-L1 positivity, indicating that GIS-High status was the only positive biomarker in a small proportion of cases (Figure 4 and Supplementary Figure S2). We observed no overlap between MSI-High and GIS-High tumors. This pattern likely reflects the distinct DNA repair pathways captured by these biomarkers: MSI-High status indicates defective DNA mismatch repair, whereas GIS-High status reflects genomic scarring associated with homologous recombination deficiency [9,24].

Several studies have evaluated HRD across diverse tumor types. However, the genomic panels and algorithms used to define HRD are varied, leading to inconsistent findings [25,26]. As a result, the true incidence of HRD and its clinicopathologic associations across diverse tumor types remain unclear. Mutational signature analysis has emerged as a more precise approach for assessing HRD. Single-base substitution signature 3 (SBS3), indel signatures such as ID6 and ID8, and rearrangement signatures such as RS3 and RS5 show strong associations with HRD [24,27,28]. However, reliable mutational signature profiling generally requires whole-genome sequencing, which is often impractical in routine clinical settings because it typically requires tumor–normal matched samples and because FFPE-derived DNA can introduce substantial sequencing artifacts [29]. Beyond sequencing-based approaches, emerging computational pathology methods have also been explored for predicting biomarkers such as MSI and TMB from routine histopathologic images. For example, Zhang et al. developed a deep learning framework that integrates whole-slide image features and nuclear segmentation to predict MSI and TMB status in gastric and colorectal cancers [30]. Although GIS prediction using such approaches remains investigational, these studies illustrate the broader movement toward integrated computational biomarker assessment.

Using the TruSight Oncology 500 (TSO500) HRD assay, which implements the genomic instability score (GIS) algorithm used in the FDA-approved Myriad myChoice CDx test, we found that GIS-High tumors occurred not only in ovarian cancer but also in a small subset of non-ovarian cancers. Because this study did not include treatment-response or clinical outcome data, these findings should be interpreted as descriptive and hypothesis-generating rather than as evidence of therapeutic benefit in non-ovarian cancers. In our ovarian cancer cohort, 41.2% (7/17) of cases were GIS-High, a proportion somewhat lower than previously reported. This difference likely reflects the inclusion of three low-grade serous carcinomas, a subtype in which GIS-High status is rare. After excluding these cases, the proportion of GIS-High ovarian cancers increased to 50.0% [22]. Notably, a squamous cell carcinoma arising in a mature cystic teratoma was unexpectedly GIS-High. Taken together, these findings suggest that HRD testing may be informative for ovarian cancer subtypes beyond high-grade serous carcinoma.

Among non-ovarian tumor types, breast invasive ductal carcinoma showed a relatively higher frequency of GIS-High tumors, consistent with previous reports [31]. Interestingly, only one of the four GIS-High breast tumors in our cohort was triple-negative breast cancer, which contrasts with the prevailing view that GIS-High status is enriched in triple-negative disease [32]. This discrepancy may reflect the small sample size. In contrast, we did not identify any GIS-High tumors among the prostate cancers in our cohort, although the number of prostate cancer cases was limited (*n* = 6). Notably, we recently detected two GIS-High prostate cancer cases at our institution, although these were not included in the present cohort. Beyond breast and prostate cancers, we identified two GIS-High lung cancers and one GIS-High hepatobiliary tract adenocarcinoma—tumor types not typically regarded as enriched for HRD. Hepatobiliary tract cancers comprise anatomically and biologically heterogeneous entities, including intrahepatic, perihilar, and distal/extrahepatic cholangiocarcinoma, as well as gallbladder carcinoma. In addition, diagnostic approaches and tissue acquisition methods may vary according to tumor location, with different combinations of imaging, endoscopic, and biopsy-based techniques [33]. Therefore, the GIS findings in hepatobiliary tract cancers should be interpreted cautiously, particularly given the small number of cases in this subgroup.

These findings suggest that GIS testing may help characterize HRD-like genomic scar patterns across selected non-ovarian tumors. Nevertheless, whether GIS-High status in non-ovarian cancers predicts sensitivity to HRD-directed therapies remains uncertain and requires validation in larger cohorts with treatment-response and outcome data. Because the GIS cutoff has been validated primarily in ovarian cancer [34,35], it is unclear whether a high GIS score carries the same biological or predictive significance in non-ovarian tumor types. It also remains uncertain whether the established GIS-High threshold of ≥42 applies to other tumor types. Therefore, GIS-High results in non-ovarian cancers should be interpreted as exploratory or potentially predictive biomarkers rather than as definitive evidence of sensitivity to HRD-directed therapies.

As expected, the GIS score was significantly associated with *BRCA1/2* mutational status (*p* < 0.001), given the central role of *BRCA1/2* in homologous recombination-mediated DNA repair [22]. However, more than half (8/14) of the GIS-High tumors did not harbor *BRCA1/2* mutations. This finding suggests that GIS-High status is not limited to *BRCA1/2*-mutated tumors and may reflect other mechanisms of homologous recombination deficiency, such as epigenetic alterations, defects in non-*BRCA* homologous recombination repair genes, or accumulated genomic scars resulting from prior HRD-related genomic instability [22]. *TP53* mutational status was also significantly associated with the GIS score (*p* < 0.001), consistent with the established link between *TP53* alteration and increased genomic instability [36]. Notably, the mutational status of other HRD-related genes, such as *ATM* and *PALB2*, was not significantly associated with the GIS score. This observation suggests that panel-based HRD assessment based solely on HRR-gene mutations may not consistently reflect downstream genomic instability, which may partly explain discrepancies in HRD prevalence estimates across tumor types when different testing approaches are used. Therefore, these findings underscore the importance of GIS-based assessment of “genomic scars”—including LOH, LST, and TAI—to define GIS-High tumors beyond mutation-only (panel-based) HRD testing.

This study has several limitations. In terms of cohort composition, the cohort was relatively small (*n* = 162), making it difficult to estimate the precise prevalence of GIS-High tumors across diverse tumor types. Subgroup analyses are also likely underpowered and should be interpreted with caution. Because testing was ordered at the discretion of treating clinicians, this cohort is subject to referral and indication bias, particularly in tumor types for which clinical suspicion of HRD may have influenced test ordering. Accordingly, the frequency of GIS-High tumors observed in this study should be interpreted as the frequency within a clinically tested cohort and should not be regarded as an estimate of population-level prevalence across tumor types. From a clinical perspective, this study did not include clinical outcome data, treatment-response analysis, or information on exposure to PARP inhibitors or other HRD-directed therapies. Therefore, the presence of GIS-High status should not be interpreted as evidence of therapeutic sensitivity, clinical actionability, or eligibility for HRD-directed treatment in non-ovarian cancers. Accordingly, our findings should be regarded as descriptive and hypothesis-generating. Future studies with larger, tumor-type-specific cohorts and integrated treatment-response and clinical outcome data will be needed to determine whether GIS-High status has predictive or therapeutic relevance in non-ovarian cancers. From a technical perspective, all cases were analyzed using a tumor-only sequencing approach, without matched normal samples. Although tumor-only bioinformatic filtering can reduce the reporting of likely germline variants, definitive discrimination between somatic and germline variants was not feasible without matched normal sequencing. Therefore, we cannot determine whether the detected *BRCA1/2* alterations were somatic or germline. For the same reason, biallelic inactivation was not systematically assessed.

Despite these limitations, we assessed the GIS scores and prevalence of GIS-High tumors across multiple cancer types using the GIS algorithm employed in the FDA-approved Myriad myChoice CDx assay. GIS-High tumors were identified in a small subset of non-ovarian cancers, supporting further investigation of GIS as an exploratory biomarker rather than establishing its therapeutic relevance in these tumor types.

## Figures and Tables

**Table 1:** Characteristics of the patient cohort.

Variables	*N* (Total = 162)	(%)
Sex		
Male	69	42.6
Female	93	57.4
Age (mean)	62.5 (0–84)	
Type of sampling		
Resection	66	40.7
Biopsy	91	56.2
Cytology	5	3.1
Tumor type		
Lung cancer	53	32.8
Breast cancer	21	13.0
Uterus (endometrial cancer)	18	11.1
Ovary cancer	17	10.5
Hepatobiliary cancer	14	8.6
Urothelial carcinoma	8	4.9
Prostate cancer	6	3.7
Brain tumor	3	1.8
Kidney cancer	3	1.8
Head and neck squamous cell carcinoma	3	1.8
Salivary gland tumor	2	1.2
Skin squamous cell carcinoma	2	1.2
Pancreas cancer	2	1.2
Pleural tumor	2	1.2
Colon cancer	2	1.2
Soft tissue tumor	2	1.2
Stomach cancer	1	0.7
Thyroid cancer	1	0.7
Uterine cervix cancer	1	0.7
Malignancy of unknown origin	1	0.7

**Table 2:** Clinicopathologic characteristics of 14 GIS-High type tumors.

No	Diagnosis	Age	Sex	GIS Score	TMB Score	MSI Score	Oncogenic Mutation	PD-L1
1	Lung, adenocarcinoma	83	M	49	7.0	2.6	*BRCA2* c.8488-1G>A, *EGFR* p.(Leu861Arg), *TERT* c.-124G>A, *TP53* p.(Arg175His), *ETV6* p.(Cys452Ter)	30 (TPS)
2	Lung, adenocarcinoma	65	M	46	13.3	1.0	*ERBB2* p.(Ile767Met), *KEAP1* p.(Gly480Trp), *TP53* p.(Pro151His), *MET* amplification	60 (TPS)
3	Hepatobiliary, adenocarcinoma	64	F	57	18.0	1.7	*ERBB2* amplification	Not tested
4	Breast, invasive ductal carcinoma (luminal type)	54	F	57	6.3	5.7	*BRCA2* p.(Cys647ValfsTer13), *PBRM1* p.(Arg1455Ter), *ZFHX3* p.(Gln758Ter), *MDM2* amplification, *FGFR4* amplification	Not tested
5	Breast, invasive ductal carcinoma (luminal type)	59	F	65	6.2	1.0	*BRCA2* p.(Pro2767HisfsTer11), *ESR1* p.(Tyr537Cys)	Not tested
6	Breast, invasive ductal carcinoma (HER2-amplified)	64	F	52	4.7	4.0	*ERBB2* amplification, *CCNE1* amplification, *ERBB3* p.(Ala232Val), *PIK3CA* p.(His1047Arg), *TP53* p.(Arg248Gln)	Not tested
7	Breast, invasive ductal carcinoma (triple-negative type)	49	F	69	7.0	1.8	*TP53* p.(Arg273His), *NF1* p.(Tyr1668Ter), *MYC* p.(Thr73Ala)	0 (CPS)
8	Ovary, squamous cell carcinoma (arising from teratoma)	66	F	76	24.4	5.2	*PIK3CA* p.(Glu545Lys), *TP53* p.(Glu336Ter), *CDH1* c.532-3_534delinsTAGATG, *CDKN2A* p.(Ser12Ter), *EZH2* p.(Lys498GlufsTer23), *LRP1B* p.(Arg414CysfsTer5), *SETD2* p.(Gln1829Ter)	Not tested
9	Ovary, high-grade serous carcinoma	72	F	76	7.1	1.0	*TP53* p.(Ile255del), *TRAF7* c.1135+1G>	Not tested
10	Ovary, high-grade serous carcinoma	69	F	55	4.0	3.0	*TP53* p.(Ala159Val)	Not tested
11	Ovary, high-grade serous carcinoma	60	F	55	4.7	0	*SMOX*::*BRCA1* Translocation, *CIC* p.(Ala1809ProfsTer24), *PPM1D* p.(Gln462Ter), *TP53* p.(Tyr234Cys)	Not tested
12	Ovary, high-grade serous carcinoma	69	F	64	6.2	2.8	*TP53* c.-29+1G>A	Not tested
13	Ovary, high-grade serous carcinoma	70	F	62	4.7	1.0	*TP53* p.(Arg213Ter), *LATS1* p.(Arg287Ter), *RECQL4* p.(Ala1066AspfsTer16), *RECQL4* p.(Ala1068LeufsTer31), *SOX17* p.(Arg67AlafsTer10)	CPS (40)
14	Ovary, high-grade serous carcinoma	67	F	62	7.4	2.9	*BRCA1* p.(Glu699Ter), *TP53* p.(Cys176Arg), *NF1* p.(Thr586ValfsTer18), *TSC2* p.(Leu236SerfsTer8)	Not tested

F: female, M: male.

## Data Availability

The original contributions presented in this study are included in the article/Supplementary Materials. Further inquiries can be directed to the corresponding authors.

## Supplementary Materials

The following supporting information can be downloaded at: https://www.mdpi.com/article/10.3390/diagnostics16121802/s1, Table S1. Selected sequencing quality control metrics. Table S2. Case-level clinicopathologic and genomic features. Table S3. Additional statistical analyses for GIS score associations. Figure S1. Tumor distribution of the patient cohort. Figure S2. Tumor-type-specific mosaic plots of biomarker distribution in the five most common tumor types.

## References

[1] Chakravarty D., Solit D.B. (2021). Clinical cancer genomic profiling. Nat. Rev. Genet..

[2] Meyerson M., Gabriel S., Getz G. (2010). Advances in understanding cancer genomes through second-generation sequencing. Nat. Rev. Genet..

[3] Budczies J., Kazdal D., Menzel M., Beck S., Kluck K., Altbürger C., Schwab C., Allgäuer M., Ahadova A., Kloor M. (2024). Tumour mutational burden: Clinical utility, challenges and emerging improvements. Nat. Rev. Clin. Oncol..

[4] Alexandrov L.B., Nik-Zainal S., Wedge D.C., Aparicio S.A., Behjati S., Biankin A.V., Bignell G.R., Bolli N., Borg A., Børresen-Dale A.L. (2013). Signatures of mutational processes in human cancer. Nature.

[5] Vanderwalde A., Spetzler D., Xiao N., Gatalica Z., Marshall J. (2018). Microsatellite instability status determined by next-generation sequencing and compared with PD-L1 and tumor mutational burden in 11,348 patients. Cancer Med..

[6] Doig K.D., Fellowes A.P., Fox S.B. (2023). Homologous Recombination Repair Deficiency: An Overview for Pathologists. Mod. Pathol..

[7] Li X., Li M., Xiang J., Zhao Z., Shang X. (2022). SEPA: Signaling entropy-based algorithm to evaluate personalized pathway activation for survival analysis on pan-cancer data. Bioinformatics.

[8] He B., Ma Y., Wang K., Bing P., Ji L., Tian G., Liu H., He P., Yang J. (2026). Microbiome-transcriptome-histology triad enhances survival risk stratification in multiple cancers. Comput. Biol. Chem..

[9] Pilié P.G., Tang C., Mills G.B., Yap T.A. (2019). State-of-the-art strategies for targeting the DNA damage response in cancer. Nat. Rev. Clin. Oncol..

[10] Kim Y.N., Gulhan D.C., Jin H., Glodzik D., Park P.J. (2024). Recent Advances in Genomic Approaches for the Detection of Homologous Recombination Deficiency. Cancer Res. Treat..

[11] Iglehart J.D., Silver D.P. (2009). Synthetic lethality—A new direction in cancer-drug development. N. Engl. J. Med..

[12] Dobzhansky T. (1946). Genetics of natural populations; recombination and variability in populations of Drosophila pseudoobscura. Genetics.

[13] Abkevich V., Timms K.M., Hennessy B.T., Potter J., Carey M.S., Meyer L.A., Smith-McCune K., Broaddus R., Lu K.H., Chen J. (2012). Patterns of genomic loss of heterozygosity predict homologous recombination repair defects in epithelial ovarian cancer. Br. J. Cancer.

[14] Birkbak N.J., Wang Z.C., Kim J.Y., Eklund A.C., Li Q., Tian R., Bowman-Colin C., Li Y., Greene-Colozzi A., Iglehart J.D. (2012). Telomeric allelic imbalance indicates defective DNA repair and sensitivity to DNA-damaging agents. Cancer Discov..

[15] Popova T., Manié E., Rieunier G., Caux-Moncoutier V., Tirapo C., Dubois T., Delattre O., Sigal-Zafrani B., Bollet M., Longy M. (2012). Ploidy and large-scale genomic instability consistently identify basal-like breast carcinomas with BRCA1/2 inactivation. Cancer Res..

[16] Fumagalli C., Betella I., Ranghiero A., Guerini-Rocco E., Bonaldo G., Rappa A., Vacirca D., Colombo N., Barberis M. (2022). In-house testing for homologous recombination repair deficiency (HRD) testing in ovarian carcinoma: A feasibility study comparing AmoyDx HRD Focus panel with Myriad myChoiceCDx assay. Pathologica.

[17] Schubart C., Tögel L., Carta M.G., Hetzner P., Helbig L., Zaglas C., Ziegler M., Stöhr R., Hölsken A., Hoyer J. (2025). Limited association between HRR gene alterations and HRD in molecular tumor board cancer samples: Who should be tested for HRD?. Int. J. Cancer.

[18] Witz A., Dardare J., Betz M., Michel C., Husson M., Gilson P., Merlin J.L., Harlé A. (2025). Homologous recombination deficiency (HRD) testing landscape: Clinical applications and technical validation for routine diagnostics. Biomark. Res..

[19] U.S. Food and Drug Administration (2018). PD-L1 IHC 22C3 pharmDx SK006 50 Tests for Use with Autostainer Link 48.

[20] Janjigian Y.Y., Shitara K., Moehler M., Garrido M., Salman P., Shen L., Wyrwicz L., Yamaguchi K., Skoczylas T., Campos Bragagnoli A. (2021). First-line nivolumab plus chemotherapy versus chemotherapy alone for advanced gastric, gastro-oesophageal junction, and oesophageal adenocarcinoma (CheckMate 649): A randomised, open-label, phase 3 trial. Lancet.

[21] Li M.M., Datto M., Duncavage E.J., Kulkarni S., Lindeman N.I., Roy S., Tsimberidou A.M., Vnencak-Jones C.L., Wolff D.J., Younes A. (2017). Standards and Guidelines for the Interpretation and Reporting of Sequence Variants in Cancer: A Joint Consensus Recommendation of the Association for Molecular Pathology, American Society of Clinical Oncology, and College of American Pathologists. J. Mol. Diagn..

[22] Konstantinopoulos P.A., Ceccaldi R., Shapiro G.I., D’Andrea A.D. (2015). Homologous Recombination Deficiency: Exploiting the Fundamental Vulnerability of Ovarian Cancer. Cancer Discov..

[23] Yamamoto H., Hirasawa A. (2021). Homologous Recombination Deficiencies and Hereditary Tumors. Int. J. Mol. Sci..

[24] Alexandrov L.B., Kim J., Haradhvala N.J., Huang M.N., Tian Ng A.W., Wu Y., Boot A., Covington K.R., Gordenin D.A., Bergstrom E.N. (2020). The repertoire of mutational signatures in human cancer. Nature.

[25] Wen H., Feng Z., Ma Y., Liu R., Ou Q., Guo Q., Shen Y., Wu X., Shao Y., Bao H. (2022). Homologous recombination deficiency in diverse cancer types and its correlation with platinum chemotherapy efficiency in ovarian cancer. BMC Cancer.

[26] Wen H., Feng Z., Ma Y., Liu R., Ou Q., Bao H., Wu X., Wu X. (2021). 67P Homologous recombination deficiency in diverse cancer types and its correlation with platinum chemotherapy efficiency in ovarian cancer. Ann. Oncol..

[27] Steele C.D., Abbasi A., Islam S.M.A., Bowes A.L., Khandekar A., Haase K., Hames-Fathi S., Ajayi D., Verfaillie A., Dhami P. (2022). Signatures of copy number alterations in human cancer. Nature.

[28] Wang C., Yin R., Dai J., Gu Y., Cui S., Ma H., Zhang Z., Huang J., Qin N., Jiang T. (2018). Whole-genome sequencing reveals genomic signatures associated with the inflammatory microenvironments in Chinese NSCLC patients. Nat. Commun..

[29] Haile S., Corbett R.D., Bilobram S., Bye M.H., Kirk H., Pandoh P., Trinh E., MacLeod T., McDonald H., Bala M. (2019). Sources of erroneous sequences and artifact chimeric reads in next generation sequencing of genomic DNA from formalin-fixed paraffin-embedded samples. Nucleic Acids Res..

[30] Zhang Y., Han J., Chen H., Hu F., Huang Y., Tian G., Zhong D., Yang J. (2025). Deep learning-based fusion of nuclear segmentation features for microsatellite instability and tumor mutational burden prediction in digestive tract cancers: A multicenter validation study. Brief. Bioinform..

[31] den Brok W.D., Schrader K.A., Sun S., Tinker A.V., Zhao E.Y., Aparicio S., Gelmon K.A. (2017). Homologous Recombination Deficiency in Breast Cancer: A Clinical Review. JCO Precis. Oncol..

[32] Ali R.M.M., McIntosh S.A., Savage K.I. (2021). Homologous recombination deficiency in breast cancer: Implications for risk, cancer development, and therapy. Genes Chromosomes Cancer.

[33] Yakub Z.A., Aebisher D., Woźnicki P., Łabądź J.M., Myśliwiec A., Dynarowicz K., Bartusik-Aebisher D. (2025). Different diagnostic methods for biliary tract cancer: A review. Adv. Radiother. Nucl. Med..

[34] González-Martín A., Pothuri B., Vergote I., DePont Christensen R., Graybill W., Mirza Mansoor R., McCormick C., Lorusso D., Hoskins P., Freyer G. (2019). Niraparib in Patients with Newly Diagnosed Advanced Ovarian Cancer. N. Engl. J. Med..

[35] Ray-Coquard I., Pautier P., Pignata S., Pérol D., González-Martín A., Berger R., Fujiwara K., Vergote I., Colombo N., Mäenpää J. (2019). Olaparib plus Bevacizumab as First-Line Maintenance in Ovarian Cancer. N. Engl. J. Med..

[36] Levine A.J. (2020). p53: 800 million years of evolution and 40 years of discovery. Nat. Rev. Cancer.

