# Supplementary material for: Genomic Instability Score Across Diverse Tumor Types Using the Illumina TruSight Oncology 500 HRD Assay
Source: Diagnostics (Basel). 2026 Jun 11;16(12):1802. doi: 10.3390/diagnostics16121802 (PMC13298436; doi:10.3390/diagnostics16121802)
Supplement: Supplementary file 1 [file diagnostics-16-01802-s001.zip › Supplementary Figure.pdf]

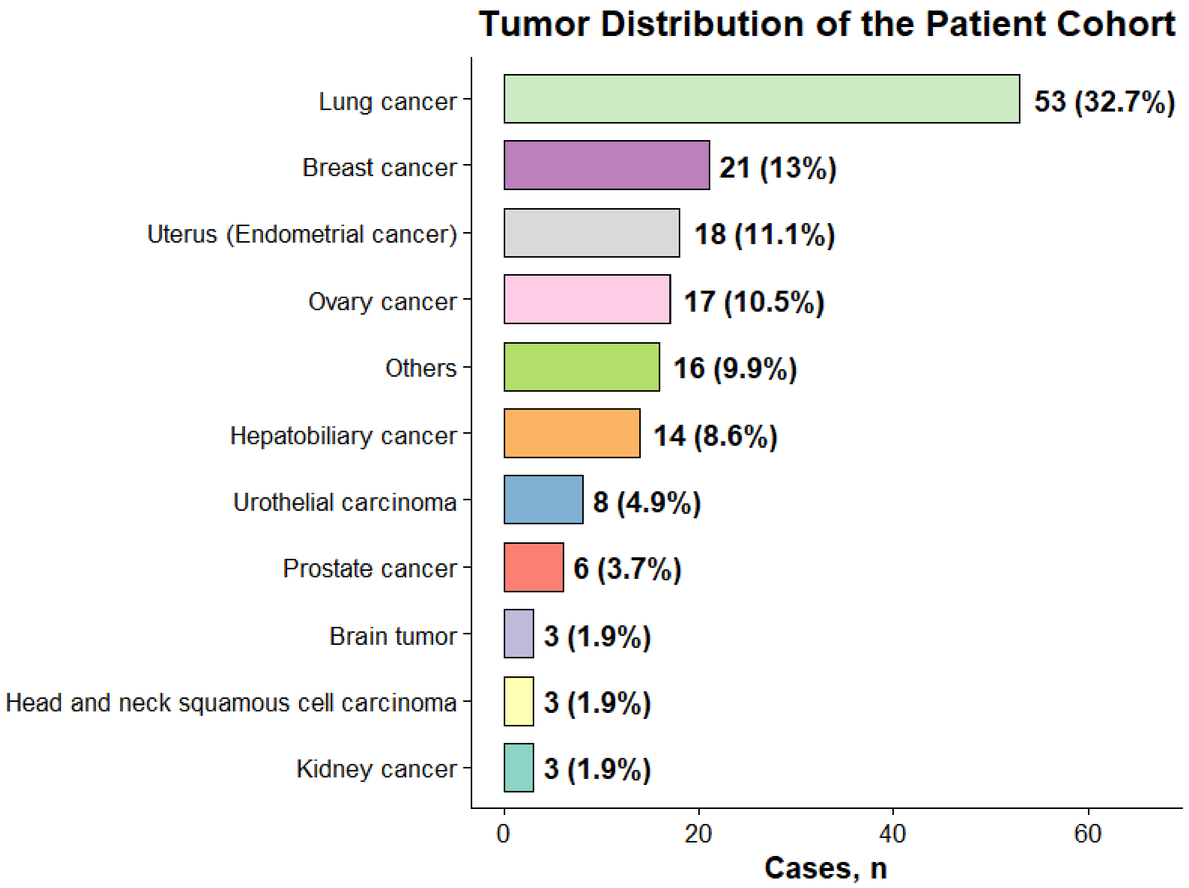

Supplementary Figure S1. Tumor distribution of the patient cohort.

Horizontal bar chart showing the distribution of tumor types included in the study cohort. Tumor types represented by two or fewer cases were grouped as “Others” to improve visualization. The number and percentage of cases are shown for each tumor group.

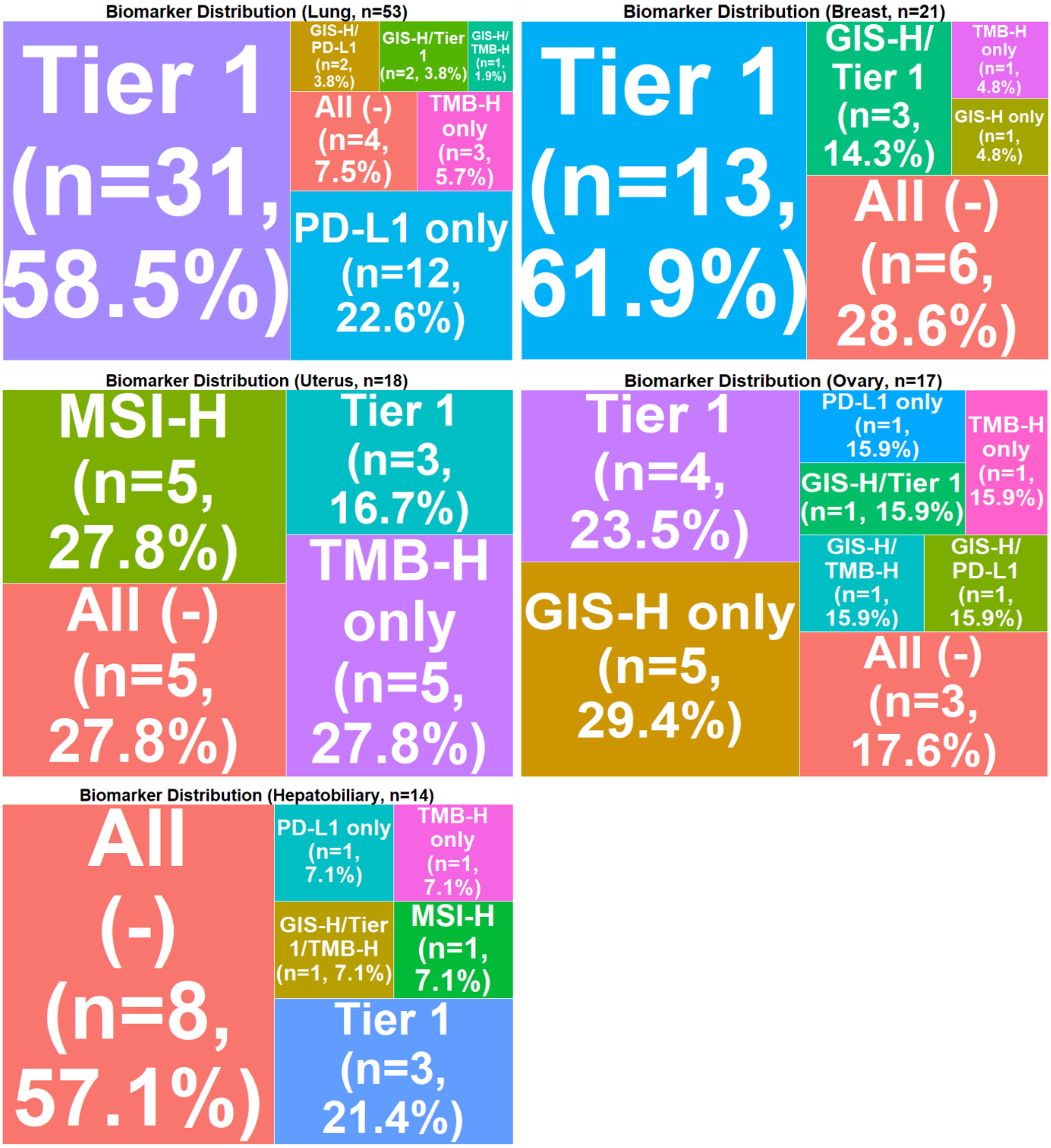

Supplementary Figure S2. Tumor-type-specific mosaic plots of biomarker distribution in the five most common tumor types.

Mosaic plots showing the tumor-type-specific breakdown of biomarker distribution and overlap patterns in the five most common tumor types in the study cohort.
