# Supplementary material for: Genomic Instability Score Across Diverse Tumor Types Using the Illumina TruSight Oncology 500 HRD Assay
Source: Diagnostics (Basel). 2026 Jun 11;16(12):1802. doi: 10.3390/diagnostics16121802 (PMC13298436; doi:10.3390/diagnostics16121802)
Supplement: Supplementary file 1 [file diagnostics-16-01802-s001.zip › Supplementary Table.pdf]

Supplementary Table S1. Selected sequencing quality control metrics

Table S1A. Summary of selected sequencing QC metrics.

Metric category	Metric	QC guideline	n	Median	IQR	Range	Values outside guideline, n/N
Run-level	PCT_PF_READS (%)	>= 80	162	86	84.1-87.3	79.6-92.5	8/162
Run-level	PCT_Q30_R1 (%)	>= 80	157	89.4	87.8-90.9	85.1-94.1	0/157
Run-level	PCT_Q30_R2 (%)	>= 80	157	87.2	85.8-89.3	82.8-92.2	0/157
DNA library QC metrics	MEDIAN_INSERT_SIZE (bp)	>= 70	162	143	133-150	78-177	0/162
DNA library QC metrics	MEDIAN_EXON_COVERAGE (Count)	>= 150	162	505	362.8-607.2	79-1096	3/162
DNA library QC metrics	PCT_EXON_50X (%)	>= 90	162	99.2	98.6-99.5	75.8-99.9	3/162
DNA library QC metrics	PCT_CHIMERIC_READS (%)	<= 8	162	1.1	0.74-1.81	0.21-7.9	0/162
DNA Library QC Metrics for MSI	USABLE_MSI_SITES (Count)	>= 40	162	106	90-114	18-122	6/162
DNA Library QC Metrics for CNV	GENE_SCALED_MAD (Count)	>= 0; <= 0.13	162	0.06	0.06-0.07	0.04-0.14	1/162
DNA Library QC Metrics for CNV	MEDIAN_BIN_COUNT_CNV_TARGET (Count)	>= 1	162	4.45	3.52-5.4	1-10.1	0/162
DNA Library QC Metrics for GIS	PCT_TARGET_HRD_50X (%)	>= 50	162	98.5	96.6-99.4	48.4-100	1/162
DNA Library QC Metrics for GIS	MEDIAN_TARGET_HRD_COVERAGE (Count)	NA	162	183.5	152-213	48-327	0/162
DNA Library QC Metrics for GIS	EXCESSIVE_TF (NA)	<= 0	162	0	0-0	0-0	0/162
RNA	MEDIAN_CV_GENE_500X (NA)	>= 0; <= 0.93	162	0.55	0.52-0.6	0.43-1.06	6/162
RNA	TOTAL_ON_TARGET_READS (Count)	>= 9,000,000	162	25,054,465	23,575,777-26,057,000	15,322,031-27,566,847	0/162
RNA	MEDIAN_INSERT_SIZE (Count)	>= 80	162	134	124.2-145	96-160	0/162
RNA	PCT_ON_TARGET_READS (%)	NA	162	86.2	82.1-88.1	53.3-92.1	0/162
RNA	SCALED_MEDIAN_GENE_COVERAGE (Count)	NA	162	5266.9	4833.5-5577.2	2348.6-6274.5	0/162
RNA	RNA_PCT_Q30_BASES (%)	NA	162	90.1	88.1-91.3	84.4-94.5	0/162

LSL, lower specification limit; USL, upper specification limit; IQR, interquartile range; HRD, homologous recombination deficiency; GIS, genomic instability score; MSI, microsatellite instability; CNV, copy number variation; TMB, tumor mutational burden. QC guideline values indicate LSL/USL values provided in the TSO500 metrics output. All 162 cases completed all analysis steps, with no failed steps or unexecuted steps recorded in the metrics output.

Table S1B. Case-level selected sequencing QC metrics.

Case No.	Completed all steps	Q30 R1, %	Q30 R2, %	Median exon coverage	Exon targets >=50x, %	HRD targets >=50x, %	Median HRD target coverage	Usable MSI sites	Gene scaled MAD	Median bin count CNV target
1	Yes	94.1	92.2	365	98	97.8	157	92	0.09	3.2
2	Yes	94.1	92.2	320	97.8	95.9	135	81	0.08	2.9
3	Yes	94.1	92.2	314	98.6	87.6	97	91	0.07	3.3
4	Yes	94.1	92.2	235	96.5	90.9	106	65	0.08	2
5	Yes	90.9	89.9	259	97.8	95.6	126	75	0.06	2.5
6	Yes	90.9	89.9	565	99	99.2	189	111	0.06	4.7
7	Yes	90.9	89.9	484	99	99.2	183	112	0.06	4
8	Yes	90.9	89.9	259	97	92.2	120	67	0.07	2.3
9	Yes	90.9	89.9	505	99.2	98.6	186	109	0.06	4.4
10	Yes	90.9	89.9	644	99.1	99.1	192	114	0.06	5.2
11	Yes	88.9	87	157	91.7	83.4	85	27	0.07	1.4
12	Yes	88.9	87	79	75.8	48.4	48	18	0.08	1
13	Yes	88.9	87	229	93.8	88.3	109	49	0.08	2
14	Yes	88.9	87	249	95.6	91.2	120	60	0.08	2.2
15	Yes	88.9	87	429	98.8	98.3	181	100	0.06	3.5
16	Yes	88.9	87	160	89.6	78.1	78	24	0.08	1.5
17	Yes	88.9	87	235	95.9	91.3	113	54	0.07	2.2
18	Yes	89.7	87.7	359	99	96.9	165	112	0.07	4.3
19	Yes	89.7	87.7	147	88.2	74.4	73	25	0.09	1.6
20	Yes	89.7	87.7	200	92.8	82.3	92	42	0.08	2.1
21	Yes	89.7	87.7	197	92.1	82.4	91	37	0.09	2
22	Yes	89.7	87.7	422	98.9	96.2	170	99	0.05	4.1
23	Yes	89.7	87.7	238	98.2	94.1	124	92	0.07	2.7
24	Yes	89.7	87.7	174	91.9	77.8	79	33	0.09	1.7
25	Yes	89.8	87.9	405	98.7	97.9	160	93	0.07	3.6
26	Yes	89.8	87.9	680	99.3	99.6	216	112	0.07	5.9
27	Yes	89.8	87.9	521	99.1	99.2	207	110	0.06	5.1
28	Yes	89.8	87.9	232	95.7	90.1	111	56	0.07	2.4
29	Yes	89.8	87.9	446	98.6	97.8	174	94	0.06	4.2
30	Yes	89.8	87.9	221	93.9	86.4	100.5	48	0.08	2.2
31	Yes	89.1	87.2	385	98.6	98.3	167	98	0.06	3.7
32	Yes	89.1	87.2	648	99.2	99.6	217	116	0.07	5.5
33	Yes	89.1	87.2	431	98.6	97.6	170	92	0.07	3.6
34	Yes	89.1	87.2	530	99.1	98.4	189	104	0.06	4.4
35	Yes	89.1	87.2	376	98.6	96.7	152	89	0.05	3.5
36	Yes	89.1	87.2	810	99.4	99.8	270	122	0.07	7.3
37	Yes	89.1	87.2	301	97.1	92	125	81	0.07	2.9
38	Yes	85.8	84.1	492	99	97.8	178	108	0.06	4.1
39	Yes	85.8	84.1	531	99	98.5	200	106	0.06	4.5
40	Yes	85.8	84.1	557	99.1	98.5	208	106	0.06	4.7
41	Yes	85.8	84.1	653	99.3	98.5	210	108	0.06	5.4
42	Yes	85.8	84.1	461	98.7	96.5	178	88	0.06	4.2
43	Yes	85.8	84.1	717	99.4	98.5	265	111	0.11	8.6
44	Yes	85.8	84.1	830	99.5	99.6	327	120	0.07	10
45	Yes	87.8	86.1	323	97.9	95.2	142	83	0.06	3
46	Yes	87.8	86.1	468	98.9	97.5	165	108	0.06	4.2
47	Yes	87.8	86.1	423	99.4	99.8	233	120	0.07	5.3
48	Yes	87.8	86.1	510	99	98	187	99	0.07	4.2
49	Yes	87.8	86.1	505	99	97.7	187	97	0.06	4.4
50	Yes	87.8	86.1	433	98.7	97.8	173	85	0.06	3.9
51	Yes	87.8	86.1	508	99.1	98.7	194	101	0.06	4.4
52	Yes	87.8	86.1	857	99.4	99.4	216	113	0.08	6.8
53	Yes	86.4	84.1	274	99.1	98	117	80	0.14	2.7
54	Yes	86.4	84.1	246	96.2	92.9	122	48	0.06	2.4

Case No.	Completed all steps	Q30 R1, %	Q30 R2, %	Median exon coverage	Exon targets >=50x, %	HRD targets >=50x, %	Median HRD target coverage	Usable MSI sites	Gene scaled MAD	Median bin count CNV target
55	Yes	86.4	84.1	642	99.4	99.7	245	120	0.05	6.9
56	Yes	86.4	84.1	160	95.6	89.8	92	92	0.09	2.3
57	Yes	86.4	84.1	450	99	98.2	174	90	0.06	4
58	Yes	86.4	84.1	527	99.2	99.4	202	104	0.05	5
59	Yes	86.4	84.1	474	98.9	99.4	215	120	0.07	6
60	Yes	85.1	82.8	540	99	98.9	175	101	0.06	4.7
61	Yes	85.1	82.8	503	99	99.2	195	108	0.05	4.8
62	Yes	85.1	82.8	986	99.3	99.6	266	116	0.08	8.8
63	Yes	85.1	82.8	686	99.3	99.1	201	108	0.07	5.8
64	Yes	85.1	82.8	567	99.2	98.3	178	95	0.07	4.7
65	Yes	85.1	82.8	673	99.5	100	305	116	0.09	8.8
66	Yes	85.1	82.8	423	98.7	97.8	173	84	0.06	3.7
67	Yes	85.1	82.8	286	98.3	96.4	131	74	0.06	2.7
68	Yes	87.2	85.4	424	99.3	99.7	208	116	0.06	5.2
69	Yes	87.2	85.4	659	99.3	99.3	215	114	0.06	5.6
70	Yes	87.2	85.4	756	99.4	99.4	232	117	0.07	6.1
71	Yes	87.2	85.4	529	99.4	99.7	219	115	0.06	5.7
72	Yes	87.2	85.4	684	99.5	99.3	213	118	0.06	6
73	Yes	87.2	85.4	475	98.9	98	182	88	0.06	4.1
74	Yes	87.2	85.4	589	99.2	99.5	227	102	0.07	5.1
75	Yes	87.2	85.4	300	97.9	95.6	140	79	0.06	2.9
76	Yes	87.8	85.8	790	99.2	99.8	258	120	0.06	6.8
77	Yes	87.8	85.8	569	99.1	99.6	232	115	0.07	5.3
78	Yes	87.8	85.8	410	99.2	99.2	190	108	0.05	4
79	Yes	87.8	85.8	280	95.7	89.7	120	64	0.07	2.4
80	Yes	87.8	85.8	298	96.8	92.8	131	67	0.06	2.7
81	Yes	87.8	85.8	556	99.3	99	196	109	0.06	4.8
82	Yes	87.8	85.8	315	96.9	93.1	136	68	0.06	2.8
83	Yes	87.8	85.8	412	98.6	96.8	168	89	0.07	3.6
84	Yes	89.4	87.3	210	96.1	91.8	113	62	0.06	2.1
85	Yes	89.4	87.3	371	99.2	99.4	196	118	0.06	4.3
86	Yes	89.4	87.3	282	98.5	97.2	161	95	0.05	2.9
87	Yes	89.4	87.3	310	97.9	94.5	138	69	0.06	2.8
88	Yes	89.4	87.3	479	99.3	99	192	106	0.05	4.3
89	Yes	89.4	87.3	489	99.5	99.7	211	115	0.05	4.7
90	Yes	89.4	87.3	567	99.4	99.5	215	107	0.05	5.2
91	Yes	89.4	86.9	701	99.3	99.6	230	115	0.07	5.8
92	Yes	89.4	86.9	615	99.3	99.5	214	110	0.06	5.1
93	Yes	89.4	86.9	651	99.2	99.4	214	112	0.07	5.1
94	Yes	89.4	86.9	646	99.5	99.2	220	121	0.06	5.7
95	Yes	89.4	86.9	566	99.6	99.9	262	118	0.06	6.1
96	Yes	89.4	86.9	404	98.7	96.1	166	98	0.06	3.5
97	Yes	90.8	89.3	612	99.8	99.2	220	109	0.06	5.7
98	Yes	90.8	89.3	420	99.8	98.5	173	104	0.07	4.1
99	Yes	90.8	89.3	643	99.8	98.5	195	109	0.07	5.4
100	Yes	90.8	89.3	527	99.6	92.2	129	84	0.13	4.2
101	Yes	90.8	89.3	609	99.7	96	143	107	0.13	5.1
102	Yes	93.1	90.7	559	99	99.4	192	118	0.06	5
103	Yes	93.1	90.7	438	99.3	99.5	188	118	0.06	4.3
104	Yes	93.1	90.7	390	99.4	99.7	209	116	0.06	4.5
105	Yes	93.1	90.7	576	99.2	98.5	180	116	0.06	4.8
106	Yes	93.1	90.7	580	99.2	99	164	115	0.07	4.7
107	Yes	93.1	90.7	513	99.3	98.9	166	114	0.06	4.2
108	Yes	93.1	90.7	617	99.3	98.4	175	118	0.06	5.1
109	Yes	93.1	90.7	388	98.9	97.3	152	109	0.07	3.4
110	Yes	89.9	88	461	98.9	99.1	195	99	0.06	4

Case No.	Completed all steps	Q30 R1, %	Q30 R2, %	Median exon coverage	Exon targets >=50x, %	HRD targets >=50x, %	Median HRD target coverage	Usable MSI sites	Gene scaled MAD	Median bin count CNV target
111	Yes	89.9	88	617	99.2	99.6	224	118	0.06	5.4
112	Yes	89.9	88	483	99	99.2	205	99	0.05	4.3
113	Yes	89.9	88	503	99.1	98.6	202	110	0.06	4.3
114	Yes	89.9	88	318	98.5	97	153	86	0.06	2.8
115	Yes	89.9	88	510	99.3	99.2	193	104	0.05	4.4
116	Yes	89.9	88	362	99.3	99.4	183	111	0.05	3.6
117	Yes	89.9	88	422	99.2	99.6	197	114	0.04	4.2
118	Yes	89.4	86.8	485	98.8	99.3	202	117	0.04	4.6
119	Yes	89.4	86.8	339	98.5	96.6	149	98	0.06	3
120	Yes	89.4	86.8	621	99.2	99	220	115	0.06	5.8
121	Yes	89.4	86.8	538	99.5	99.7	228	117	0.05	5.9
122	Yes	89.4	86.8	218	96.2	88.9	102	49	0.11	2.1
123	Yes	89.4	86.8	602	99.3	99.4	209	114	0.06	5.4
124	Yes	89.4	86.8	784	99.5	99.1	213	111	0.11	6.3
125	Yes	89.4	86.8	904	99.5	99.6	255	118	0.07	10.1
126	Yes	92.3	89.8	515	98.8	99.6	218	116	0.06	4.9
127	Yes	92.3	89.8	326	98.3	96.8	146	83	0.06	3.1
128	Yes	92.3	89.8	558	99.1	99.2	212	97	0.07	4.9
129	Yes	92.3	89.8	1096	99.5	99.8	297	121	0.08	9.7
130	Yes	92.3	89.8	461	98.8	98	178	86	0.07	3.9
131	Yes	92.3	89.8	496	99	98.7	184	99	0.07	4.1
132	Yes	92.3	89.8	278	99.3	98.4	132	118	0.07	3.6
133	Yes	92.3	89.8	146	97.3	93	94	98	0.07	1.9
134	Yes	NA	NA	710	99.8	99.1	254	100	0.07	6.8
135	Yes	NA	NA	564	99.8	96.6	156	90	0.08	4.6
136	Yes	NA	NA	653	99.8	96.1	146	95	0.08	5.8
137	Yes	NA	NA	590	99.8	97.8	171	95	0.08	5
138	Yes	NA	NA	533	99.7	97.3	165	80	0.08	4.6
139	Yes	91.6	90.1	600	99.8	99.6	224	115	0.06	5.5
140	Yes	91.6	90.1	709	99.8	99	227	98	0.08	6.4
141	Yes	91.6	90.1	673	99.8	99.1	215	106	0.07	5.8
142	Yes	91.6	90.1	583	99.7	93.9	144	90	0.08	5.4
143	Yes	91.6	90.1	552	99.8	98.5	174	102	0.07	4.9
144	Yes	91.6	90.1	476	99.8	98.1	163	91	0.07	4.2
145	Yes	91.6	90.1	560	99.8	98.2	183	88	0.08	4.7
146	Yes	91.6	90.1	723	99.8	99.7	270	109	0.07	6.6
147	Yes	90.9	89.3	647	99.8	98.9	199	108	0.07	5.7
148	Yes	90.9	89.3	530	99.7	95.9	154	103	0.07	4.6
149	Yes	90.9	89.3	621	99.8	98	184	109	0.07	5.2
150	Yes	90.9	89.3	701	99.8	99.5	249	116	0.06	6.7
151	Yes	90.9	89.3	584	99.8	98.6	189	113	0.07	4.9
152	Yes	90.9	89.3	579	99.9	98.9	192	109	0.07	4.6
153	Yes	90.9	89.3	427	99.8	98.1	150	112	0.06	3.9
154	Yes	90.9	89.3	691	99.8	99.3	226	110	0.07	6.5
155	Yes	87.8	85	642	99.8	99.7	248	118	0.05	6
156	Yes	87.8	85	540	99.8	98.5	168	100	0.07	4.7
157	Yes	87.8	85	299	99.4	99.6	188	111	0.07	3.8
158	Yes	87.8	85	516	99.8	98.7	175	108	0.06	4.7
159	Yes	87.8	85	645	99.8	99.6	250	115	0.06	6.3
160	Yes	87.8	85	564	99.7	99.1	215	107	0.06	5.5
161	Yes	87.8	85	327	99.7	99.5	171	106	0.07	3.6
162	Yes	87.8	85	635	99.8	98.8	191	106	0.07	5.5

Only selected de-identified QC metrics relevant to run quality, coverage depth, GIS/HRD assessment, MSI assessment, and CNV analysis are shown. Internal accession numbers, run identifiers, and nonessential workflow fields were omitted.

Supplementary Table S2. Case-level clinicopathologic and genomic features

Table S2A. Clinical information, GIS, and screening biomarkers (GIS/TMB/MSI).

Case No.	Organ (primary site)	Age	Sex	Sampling method	Sampling site	Diagnosis	GIS score	Targetable alteration	GIS-High	TMB-High	MSI-High
1	Ovary	69	Female	resection	ovary	high-grade serous carcinoma	39	None	No	No	No
2	Uterus	63	Female	resection	uterus	endometrioid carcinoma	3	None	No	Yes	Yes
3	Prostate	67	Male	biopsy	prostate	adenocarcinoma	27	BRCA2 p.(Ser1205LeufsTer4), BRCA2 p.(Arg2494Ter)	No	Yes	No
4	Ureter	76	Male	biopsy	kidney	urothelial carcinoma	17	None	No	Yes	No
5	Ureter	72	Male	biopsy	urinary bladder	urothelial carcinoma	26	ERBB2 amplification	No	Yes	No
6	Brain	56	Male	resection	brain	glioma	6	None	No	Yes	No
7	Uterus	68	Female	resection	uterus	endometrioid carcinoma	7	None	No	Yes	No
8	Hepatobiliary	66	Male	resection	bile duct	cholangiocarcinoma	11	ERBB2 amplification	No	No	No
9	Uterus	55	Female	resection	uterus	endometrioid carcinoma	4	PPP2R1A p.(Pro179Arg)	No	No	No
10	Breast	53	Female	biopsy	breast	invasive ductal carcinoma	14	PIK3CA p.(Glu545Lys), PTEN p.(Tyr27Cys)	No	No	No
11	Lung	78	Female	biopsy	lung	adenocarcinoma	8	MET exon 14 skipping	No	No	No
12	Uterus	54	Female	resection	uterus	endometrioid carcinoma	10	None	No	Yes	Yes
13	Lung	75	Female	biopsy	lung	adenocarcinoma	17	MET exon 14 skipping	No	No	No
14	Pleura	64	Female	biopsy	pleura	synovial sarcoma	2	None	No	No	No
15	Kidney	71	Male	resection	kidney	clear cell renal cell carcinoma	8	None	No	No	No
16	Lung	55	Male	biopsy	lymph node	adenocarcinoma	23	KIF5B::RET fusion	No	Yes	No
17	Lung	78	Male	resection	lung	adenocarcinoma	24	None	No	Yes	No
18	Lung	56	Male	biopsy	lymph node	adenocarcinoma	26	MET amplification	No	No	No
19	Uterus	51	Female	biopsy	uterus	uterine serous carcinoma	18	None	No	No	No
20	Head and neck	58	Male	biopsy	head and neck	squamous cell carcinoma	23	None	No	No	No
21	Ovary	69	Female	resection	ovary	high-grade serous carcinoma	64	None	Yes	No	No
22	Head and neck	54	Male	biopsy	liver	squamous cell carcinoma	0	None	No	No	No
23	Lung	57	Male	biopsy	lymph node	adenocarcinoma	35	None	No	Yes	No
24	Uterus	53	Female	resection	uterus	endometrioid carcinoma	1	None	No	Yes	No
25	Lung	78	Male	biopsy	lymph node	adenocarcinoma	19	None	No	No	No
26	Lung	72	Male	biopsy	lymph node	adenocarcinoma	1	None	No	No	No
27	Hepatobiliary	60	Male	biopsy	bile duct	cholangiocarcinoma	13	None	No	No	No
28	Uterus	64	Female	resection	uterus	clear cell carcinoma	1	None	No	No	No
29	Lung	55	Male	resection	brain	adenocarcinoma	20	None	No	No	No
30	Ovary	63	Female	biopsy	breast	high-grade serous carcinoma	38	CCNE1 amplification	No	Yes	No
31	Kidney	0	Male	biopsy	liver	neuroblastoma	6	None	No	No	No
32	Lung	57	Male	biopsy	lung	adenocarcinoma	38	MET amplification	No	Yes	No
33	Hepatobiliary	73	Male	biopsy	common bile duct	adenocarcinoma	2	None	No	Yes	Yes
34	Lung	75	Female	biopsy	lymph node	adenocarcinoma	16	None	No	No	No
35	Small intestine	56	Female	resection	small intestine	gastrointestinal stromal tumor	6	KIT p.(Met552_Lys558del)	No	No	No
36	Breast	49	Female	resection	breast	invasive ductal carcinoma	69	None	Yes	No	No
37	Ovary	69	Female	resection	colon	high-grade serous carcinoma	55	None	Yes	No	No
38	Ovary	61	Female	resection	ovary	high-grade serous carcinoma	25	None	No	No	No
39	Lung	84	Female	biopsy	lung	squamous cell carcinoma	17	MET exon 14 skipping	No	No	No
40	Breast	48	Female	biopsy	breast	invasive ductal carcinoma	22	None	No	No	No
41	MUO	56	Female	resection	lung	adenocarcinoma	31	None	No	No	No
42	Pleura	73	Male	biopsy	lung	mesothelioma	6	None	No	No	No
43	Ovary	60	Female	resection	ovary	carcinosarcoma	28	PPP2R1A p.(Pro179Arg)	No	No	No
44	Uterus	40	Female	resection	uterus	endometrioid carcinoma	3	None	No	No	No
45	Breast	42	Female	biopsy	liver	invasive ductal carcinoma	1	None	No	No	No
46	Lung	72	Male	biopsy	lung	squamous cell carcinoma	39	None	No	No	No
47	Lung	81	Female	cytology	ascites	adenocarcinoma	5	EGFR exon 19 deletion	No	No	No
48	Uterus	66	Female	resection	uterus	endometrioid carcinoma	5	None	No	No	No
49	Hepatobiliary	55	Female	biopsy	liver	cholangiocarcinoma	14	None	No	No	No
50	Lung	73	Male	biopsy	soft tissue	adenocarcinoma	10	None	No	No	No
51	Hepatobiliary	70	Male	biopsy	bile duct	cholangiocarcinoma	10	None	No	No	No
52	Lung	78	Female	biopsy	lymph node	adenocarcinoma	30	MET exon 14 skipping	No	No	No
53	Lung	78	Female	biopsy	lymph node	adenocarcinoma	27	MET exon 14 skipping	No	No	No
54	Breast	52	Female	resection	lymph node	invasive lobular carcinoma	5	PIK3CA p.(His1047Arg)	No	No	No
55	Skin	58	Female	biopsy	lymph node	melanoma	14	NRF1::BRAF fusion	No	No	No

Case No.	Organ (primary site)	Age	Sex	Sampling method	Sampling site	Diagnosis	GIS score	Targetable alteration	GIS-High	TMB-High	MSI-High
56	Uterus	51	Female	resection	pelvic cavity	endometrioid carcinoma	2	None	No	Yes	No
57	Lung	71	Male	biopsy	lymph node	adenocarcinoma	11	KRAS p.(Gly12Val)	No	No	No
58	Lung	65	Male	biopsy	lung	adenocarcinoma	46	ERBB2 p.(Ile767Met)	Yes	Yes	No
59	Prostate	76	Male	biopsy	prostate	adenocarcinoma	19	None	No	No	No
60	Lung	50	Male	biopsy	lung	adenocarcinoma with sarcomatoid features	22	None	No	No	No
61	Ovary	60	Female	resection	ovary	low-grade serous carcinoma	3	None	No	No	No
62	Hepatobiliary	72	Male	biopsy	bile duct	adenocarcinoma	9	None	No	No	No
63	Lung	51	Male	biopsy	lymph node	adenocarcinoma with sarcomatoid features	25	None	No	Yes	No
64	Lung	83	Male	biopsy	lymph node	adenocarcinoma	49	EGFR p.(Leu861Arg)	Yes	No	No
65	Uterus	80	Female	resection	uterus	endometrioid carcinoma	20	CCNE1 amplification	No	No	No
66	Hepatobiliary	64	Female	resection	gallbladder	adenocarcinoma	57	ERBB2 amplification	Yes	Yes	No
67	Salivary gland	56	Female	resection	salivary gland	adenoid cystic carcinoma	11	None	No	No	No
68	Breast	42	Female	biopsy	liver	invasive ductal carcinoma	27	AKT1 p.(Glu17Lys), BRAF p.(Val600Glu)	No	No	No
69	Breast	60	Female	biopsy	liver	invasive ductal carcinoma	13	ESR1 p.(Leu536Pro), PIK3CA p.(His1047Arg)	No	No	No
70	Ureter	59	Male	resection	kidney	urothelial carcinoma	27	None	No	No	No
71	Ovary	77	Female	resection	ovary	clear cell carcinoma	20	None	No	Yes	Yes
72	Ureter	77	Male	biopsy	urinary bladder	urothelial carcinoma	26	FGFR3 p.(Tyr373Cys)	No	Yes	No
73	Hepatobiliary	70	Female	resection	bile duct	cholangiocarcinoma	5	None	No	No	No
74	Lung	55	Female	biopsy	pleura	adenocarcinoma	10	EGFR exon 19 deletion	No	Yes	No
75	Breast	45	Female	biopsy	liver	invasive ductal carcinoma	21	ERBB2 amplification	No	No	No
76	Ovary	57	Female	resection	pelvic cavity	low-grade serous carcinoma	4	None	No	No	No
77	Breast	51	Female	biopsy	breast	invasive ductal carcinoma	10	PTEN p.(Gly251Val)	No	No	No
78	Breast	44	Female	biopsy	breast	invasive ductal carcinoma	19	None	No	No	No
79	Prostate	75	Male	biopsy	prostate	adenocarcinoma	22	None	No	No	No
80	Hepatobiliary	83	Male	biopsy	liver	cholangiocarcinoma	36	FGFR2::SLMAP fusion	No	No	No
81	Lung	56	Female	biopsy	lymph node	adenosquamous cell carcinoma	17	EGFR p.(Leu858Arg)	No	No	No
82	Lung	73	Female	biopsy	lymph node	adenocarcinoma	15	CCDC6::RET fusion	No	No	No
83	Uterus	36	Female	resection	uterus	endometrioid carcinoma	1	None	No	No	No
84	Ureter	66	Female	biopsy	neck	urothelial carcinoma	16	None	No	No	No
85	Uterus	67	Female	resection	uterus	endometrioid carcinoma	2	AKT1 p.(Glu17Lys)	No	No	No
86	Skin	69	Male	biopsy	skin	squamous cell carcinoma	19	None	No	Yes	No
87	Lung	79	Male	biopsy	soft tissue	adenocarcinoma	13	None	No	Yes	No
88	Lung	76	Male	biopsy	lung	adenocarcinoma	0	None	No	No	No
89	Ovary	36	Female	resection	ovary	clear cell carcinoma	12	None	No	Yes	Yes
90	Head and neck	67	Male	biopsy	head and neck	squamous cell carcinoma	0	None	No	No	No
91	Hepatobiliary	71	Male	biopsy	bile duct	small cell carcinoma	28	None	No	Yes	No
92	Pancreas	60	Female	biopsy	pancreas	adenocarcinoma	28	KRAS p.(Gly12Val)	No	No	No
93	Ureter	55	Male	resection	kidney	urothelial carcinoma	8	HRAS p.(Lys117Asn)	No	No	No
94	Lung	57	Male	resection	lung	adenocarcinoma	36	None	No	Yes	No
95	Breast	54	Female	biopsy	liver	invasive ductal carcinoma	57	BRCA2 p.(Cys647ValfsTer13)	Yes	No	No
96	Lung	74	Female	biopsy	lung	adenocarcinoma	36	EGFR p.(Leu858Arg)	No	No	No
97	Hepatobiliary	68	Male	biopsy	bile duct	cholangiocarcinoma	10	None	No	No	No
98	Breast	52	Female	resection	breast	invasive ductal carcinoma	25	None	No	Yes	No
99	Lung	66	Male	biopsy	lymph node	adenocarcinoma	6	KRAS p.(Gly12Arg)	No	No	No
100	Thyroid	71	Male	biopsy	thyroid	anaplastic thyroid carcinoma	19	NRAS p.(Gln61Arg)	No	No	No
101	Breast	60	Female	biopsy	liver	invasive ductal carcinoma	32	ESR1::N4BP2 fusion, ESR1 p.(Tyr537Cys), PIK3CA p.(Glu542Lys)	No	No	No
102	Ureter	69	Male	biopsy	lung	urothelial carcinoma	13	None	No	No	No
103	Ovary	70	Female	resection	ovary	high-grade serous carcinoma	62	None	Yes	No	No
104	Prostate	67	Male	biopsy	prostate	adenocarcinoma	14	None	No	No	No
105	Lung	49	Male	resection	bone	adenocarcinoma	24	EGFR exon 19 deletion	No	Yes	No
106	Lung	52	Female	biopsy	lung	adenocarcinoma	0	None	No	No	No
107	Ovary	70	Female	resection	ovary	clear cell carcinoma	9	CCNE1 amplification	No	No	No
108	Brain	69	Male	biopsy	brain	glioblastoma	29	None	No	Yes	No
109	Uterus	73	Female	resection	uterus	endometrioid carcinoma	5	None	No	Yes	No
110	Brain	81	Male	resection	brain	glioblastoma	17	None	No	No	No
111	Ovary	66	Female	resection	ovary	squamous cell carcinoma	76	None	Yes	Yes	No
112	Breast	43	Female	resection	breast	invasive ductal carcinoma	3	AKT1 p.(Glu17Lys)	No	No	No

Case No.	Organ (primary site)	Age	Sex	Sampling method	Sampling site	Diagnosis	GIS score	Targetable alteration	GIS-High	TMB-High	MSI-High
113	Pancreas	60	Female	resection	pancreas	adenocarcinoma	6	KRAS p.(Gly12Asp)	No	No	No
114	Lung	61	Male	cytology	lymph node	adenocarcinoma	1	None	No	No	No
115	Lung	58	Female	resection	lung	adenocarcinoma	8	EGFR exon 19 deletion	No	No	No
116	Ovary	72	Female	resection	ovary	high-grade serous carcinoma	76	None	Yes	No	No
117	Lung	73	Male	cytology	ascites	adenocarcinoma	22	KRAS p.(Gly12Val)	No	No	No
118	Breast	64	Female	biopsy	liver	invasive ductal carcinoma	28	PIK3CA p.(Glu545Lys)	No	No	No
119	Uterine cervix	44	Female	resection	lymph node	adenocarcinoma, HPV-associated	11	None	No	Yes	No
120	Hepatobiliary	84	Female	biopsy	lung	adenocarcinoma	25	None	No	No	No
121	Breast	64	Female	biopsy	liver	invasive ductal carcinoma	52	ERBB2 amplification, PIK3CA p.(His1047Arg)	Yes	No	No
122	Uterus	61	Female	resection	uterus	endometrioid carcinoma	1	None	No	Yes	No
123	Salivary gland	74	Male	resection	parotid gland	adenoid cystic carcinoma	1	None	No	No	No
124	Prostate	74	Male	biopsy	prostate	adenocarcinoma	0	None	No	No	No
125	Ovary	50	Female	resection	ovary	low-grade serous carcinoma	0	BRAF p.(Val600Glu)	No	No	No
126	Kidney	5	Female	resection	omentum	nephroblastoma	5	None	No	No	No
127	Lung	51	Male	biopsy	lung	adenocarcinoma	5	None	No	No	No
128	Colon	81	Male	biopsy	liver	adenocarcinoma	24	None	No	Yes	No
129	Colon	73	Male	biopsy	colon	adenocarcinoma	20	None	No	Yes	Yes
130	Soft tissue	41	Female	biopsy	lymph node	Ewing sarcoma	5	None	No	No	No
131	Ovary	60	Female	biopsy	liver	high-grade serous carcinoma	55	SMOX::BRCA1 fusion	Yes	No	No
132	Uterus	60	Female	biopsy	peritoneum	endometrioid carcinoma	1	PPP2R1A p.(Arg183Gln)	No	Yes	Yes
133	Lung	75	Female	biopsy	pleura	adenocarcinoma	5	None	No	No	No
134	Lung	67	Male	biopsy	lymph node	adenocarcinoma	13	None	No	No	No
135	Lung	71	Female	biopsy	lymph node	adenocarcinoma	11	EGFR p.(Leu858Arg)	No	No	No
136	Ovary	67	Female	resection	pelvic mass	high-grade serous carcinoma	62	BRCA1 p.(Glu699Ter)	Yes	No	No
137	Uterus	77	Female	resection	uterus	endometrioid carcinoma	0	None	No	Yes	Yes
138	Prostate	69	Male	biopsy	prostate	adenocarcinoma	19	None	No	No	No
139	Hepatobiliary	74	Female	resection	gallbladder	adenocarcinoma	22	None	No	No	No
140	Lung	68	Male	resection	lung	adenocarcinoma	14	None	No	No	No
141	Lung	66	Male	biopsy	lung	adenocarcinoma	28	None	No	Yes	No
142	Lung	40	Male	biopsy	lung	adenocarcinoma	2	PWWP2A::ROS1 fusion	No	No	No
143	Uterus	55	Female	resection	lung	endometrioid carcinoma	0	None	No	Yes	Yes
144	Breast	50	Female	resection	breast	invasive ductal carcinoma	22	PTEN p.(Asp92Val), PTEN p.(Tyr155Cys)	No	No	No
145	Ureter	71	Male	resection	bladder	urothelial carcinoma	20	None	No	Yes	No
146	Breast	59	Female	biopsy	liver	invasive mucinous carcinoma	65	BRCA2 p.(Pro2767HisfsTer11), ESR1 p.(Tyr537Cys)	Yes	No	No
147	Lung	63	Male	resection	lung	adenocarcinoma	14	None	No	No	No
148	Breast	73	Female	resection	bx	invasive ductal carcinoma	38	FGFR1 amplification	No	No	No
149	Stomach	73	Female	resection	stomach	gastrointestinal stromal tumor	3	KIT p.(Val559Asp)	No	No	No
150	Breast	49	Female	resection	lung	invasive ductal carcinoma	14	None	No	No	No
151	Breast	44	Female	resection	breast	invasive mucinous carcinoma	5	None	No	No	No
152	Hepatobiliary	73	Male	biopsy	liver	adenocarcinoma	14	None	No	No	No
153	Lung	49	Male	biopsy	lymph node	adenocarcinoma	26	SLC3A2::NRG1 fusion	No	No	No
154	Lung	56	Male	biopsy	lymph node	adenocarcinoma	1	KIF5B::RET fusion	No	No	No
155	Lung	54	Male	biopsy	lymph node	adenocarcinoma	19	MET amplification	No	Yes	No
156	Lung	58	Female	biopsy	lymph node	adenocarcinoma	11	CD74::ROS1 fusion	No	No	No
157	Lung	59	Female	biopsy	lung	adenocarcinoma	10	EGFR p.(Val769_Asp770insGlyValVal)	No	No	No
158	Lung	64	Male	biopsy	lung	adenocarcinoma	18	None	No	No	No
159	Lung	60	Female	resection	lung	adenocarcinoma	8	EML4::ALK fusion	No	No	No
160	Lung	63	Male	resection	lung	adenocarcinoma	12	EGFR p.(Leu858Arg)	No	No	No
161	Lung	70	Female	cytology	lymph node	adenocarcinoma	13	TPM3::ROS1 fusion	No	No	No
162	Lung	62	Female	cytology	lymph node	adenocarcinoma	22	EML4::ALK fusion	No	No	No

Table S2B. Genomic biomarkers (TMB/MSI/HRD-related genes) and PD-L1 results.

Case No.	TMB	MSI	BRCA1/2 pathogenic alteration	Other HRD-related gene alteration	TP53 mutation	PD-L1 TPS	PD-L1 CPS
1	3.1	0.96	No	No	Yes	Not available	Not available
2	34.3	15.32	No	No	No	Not available	Not available
3	11.0	5.60	BRCA2 p.(Ser1205LeufsTer4), BRCA2 p.(Arg2494Ter)	No	Yes	Not available	Not available
4	14.8	4.08	No	No	No	Not available	Not available
5	14.5	0.00	No	No	Yes	Not available	Not available
6	10.2	1.00	No	No	No	Not available	Not available
7	395.0	4.17	No	No	Yes	Not available	90
8	6.3	2.70	No	No	Yes	Not available	Not available
9	2.6	0.00	No	No	Yes	Not available	Not available
10	4.2	3.70	No	PALB2 p.(Lys85AsnfsTer12)	No	Not available	Not available
11	4.8	0.00	No	No	Yes	0	Not available
12	33.9	30.90	No	No	No	Not available	10
13	5.0	2.70	No	No	No	100	Not available
14	3.1	0.00	No	No	No	Not available	Not available
15	2.4	1.10	No	No	No	Not available	Not available
16	11.7	3.00	No	No	Yes	95	Not available
17	16.5	3.20	No	PALB2 p.(Ser792Ter)	Yes	5	Not available
18	9.4	0.90	No	No	Yes	0	Not available
19	4.7	2.70	No	No	Yes	Not available	Not available
20	3.2	0.00	No	No	Yes	0	0
21	6.2	2.75	No	No	Yes	Not available	Not available
22	2.4	4.30	No	No	No	Not available	Not available
23	28.7	0.00	No	No	Yes	0	Not available
24	204.5	4.10	No	No	No	Not available	10
25	3.1	5.17	No	No	Yes	1	Not available
26	2.4	1.00	No	ATM p.(Lys2749Ile)	No	1	Not available
27	3.9	0.00	No	No	No	Not available	Not available
28	1.6	1.10	No	No	No	Not available	Not available
29	3.9	0.80	No	No	No	0	Not available
30	10.4	3.70	No	No	Yes	Not available	Not available
31	5.6	2.40	No	No	No	Not available	Not available
32	22.0	0.00	No	No	Yes	50	Not available
33	56.5	15.09	No	No	Yes	Not available	Not available
34	3.9	0.93	No	BRIP1 p.(Lys752ArgfsTer12)	Yes	90	Not available
35	1.6	1.14	No	No	No	Not available	Not available
36	7.0	1.80	No	No	Yes	0	0
37	4.0	3.03	No	No	Yes	Not available	Not available
38	4.7	0.83	No	No	Yes	1	5
39	5.5	1.20	No	No	Yes	Not available	Not available
40	4.7	1.85	No	No	No	Not available	Not available
41	1.6	1.67	No	No	Yes	Not available	Not available
42	2.4	2.02	No	No	No	0	Not available
43	3.1	3.09	No	No	Yes	Not available	Not available
44	4.7	2.35	No	No	No	Not available	Not available
45	3.9	1.98	No	No	No	Not available	Not available
46	5.5	2.65	No	No	Yes	15	Not available
47	7.8	6.25	No	No	Yes	0	Not available
48	8.0	0.00	No	No	No	Not available	Not available
49	3.1	1.67	No	No	No	Not available	Not available
50	6.5	8.70	No	No	Yes	100	Not available
51	8.6	0.00	No	No	No	Not available	Not available
52	7.8	0.92	No	No	Yes	80	Not available
53	7.1	0.99	No	No	Yes	80	Not available
54	3.9	0.93	No	No	No	Not available	Not available
55	3.9	1.72	No	No	No	Not available	Not available
56	11.8	3.85	No	No	No	Not available	Not available
57	6.3	2.78	No	No	Yes	Not available	Not available
58	13.3	1.05	No	No	Yes	60	Not available
59	5.5	0.86	No	No	Yes	Not available	Not available

Case No.	TMB	MSI	BRCA1/2 pathogenic alteration	Other HRD-related gene alteration	TP53 mutation	PD-L1 TPS	PD-L1 CPS
60	9.4	2.38	No	No	Yes	40	Not available
61	3.1	3.45	No	No	No	Not available	Not available
62	9.5	4.05	No	No	Yes	Not available	Not available
63	12.5	0.00	No	No	Yes	80	Not available
64	7.0	2.56	BRCA2 c.8488-1G>A	No	Yes	30	Not available
65	6.3	1.67	No	No	Yes	Not available	Not available
66	18.0	1.74	BRCA2 p.(Gln499Ter)	No	Yes	Not available	Not available
67	1.6	0.00	No	No	No	0	0
68	3.9	1.14	No	No	Yes	0	Not available
69	7.1	0.98	No	ATM p.(Gly2765Ser)	No	Not available	Not available
70	4.7	2.50	No	No	Yes	Not available	Not available
71	36.4	34.18	No	No	Yes	Not available	Not available
72	11.0	1.74	No	No	No	Not available	Not available
73	7.6	2.78	No	No	Yes	Not available	0
74	10.5	1.56	No	No	Yes	1	Not available
75	4.0	0.00	No	No	Yes	Not available	Not available
76	2.4	1.61	No	No	No	Not available	Not available
77	0.8	0.92	No	No	No	Not available	Not available
78	4.0	1.47	No	No	No	Not available	Not available
79	4.7	4.49	No	No	No	Not available	Not available
80	3.1	0.85	No	No	Yes	Not available	Not available
81	7.1	3.16	No	No	Yes	40	Not available
82	7.1	2.90	No	No	Yes	15	Not available
83	4.7	0.94	No	No	No	Not available	Not available
84	8.6	1.74	No	No	Yes	Not available	Not available
85	3.9	3.74	No	No	No	1	1
86	10.2	4.35	No	No	Yes	Not available	Not available
87	21.2	2.78	No	No	No	0	Not available
88	3.9	1.79	No	No	Yes	0	Not available
89	33.6	35.54	No	No	No	1	1
90	1.6	2.54	No	No	No	0	0
91	14.9	3.06	No	No	Yes	Not available	Not available
92	4.7	3.39	No	No	Yes	Not available	Not available
93	0.8	3.39	No	No	No	Not available	Not available
94	28.2	2.59	No	No	No	0	Not available
95	6.3	5.70	BRCA2 p.(Cys647ValfsTer13)	No	No	Not available	Not available
96	3.9	3.48	No	No	No	1	Not available
97	3.9	0.00	No	No	Yes	Not available	Not available
98	11.0	5.08	No	No	Yes	Not available	Not available
99	3.9	0.00	No	No	Yes	0	Not available
100	3.9	1.01	No	No	Yes	Not available	Not available
101	4.7	1.71	No	No	No	Not available	Not available
102	4.7	1.69	No	No	No	Not available	Not available
103	4.7	1.01	No	No	Yes	Not available	40
104	7.1	0.91	No	No	No	Not available	Not available
105	11.8	3.49	No	No	Yes	Not available	Not available
106	0.0	3.85	No	No	No	1	Not available
107	8.6	0.90	No	No	No	Not available	Not available
108	20.5	0.00	No	No	Yes	Not available	Not available
109	12.5	1.75	No	No	No	Not available	Not available
110	4.7	2.61	No	No	Yes	Not available	Not available
111	24.4	5.17	No	No	Yes	Not available	Not available
112	2.4	1.20	No	No	No	Not available	Not available
113	3.9	2.56	No	No	Yes	Not available	Not available
114	8.8	4.08	No	No	Yes	1	Not available
115	4.7	0.90	No	No	No	5	Not available
116	7.1	1.03	No	No	Yes	Not available	Not available
117	5.5	3.60	No	No	No	0	Not available
118	5.5	0.85	No	No	No	Not available	Not available
119	14.9	2.48	No	No	No	Not available	Not available
120	7.1	2.33	No	No	No	1	Not available

Case No.	TMB	MSI	BRCA1/2 pathogenic alteration	Other HRD-related gene alteration	TP53 mutation	PD-L1 TPS	PD-L1 CPS
121	4.7	4.04	No	No	Yes	Not available	Not available
122	152.8	0.87	No	No	No	Not available	40
123	3.9	0.00	No	No	No	0	0
124	0.0	2.04	No	No	No	Not available	Not available
125	3.9	0.93	No	No	No	Not available	Not available
126	1.6	5.00	No	No	No	Not available	Not available
127	2.3	1.11	No	No	No	50	Not available
128	10.1	2.04	No	No	Yes	Not available	Not available
129	27.3	29.25	No	No	No	Not available	Not available
130	1.6	2.22	No	No	No	Not available	Not available
131	4.7	0.00	SMOX::BRCA1 fusion	No	Yes	Not available	Not available
132	62.4	25.27	No	No	No	Not available	Not available
133	1.6	6.32	No	No	No	65	Not available
134	7.0	5.26	No	No	Yes	0	Not available
135	6.2	2.50	No	No	Yes	0	Not available
136	7.4	2.91	BRCA1 p.(Glu699Ter)	No	Yes	Not available	Not available
137	16.4	32.08	No	No	No	Not available	5
138	5.5	2.27	No	No	No	Not available	Not available
139	2.3	4.59	No	No	Yes	Not available	Not available
140	2.3	1.72	No	No	Yes	Not available	Not available
141	21.0	5.31	VAT1::BRCA1 fusion	No	Yes	1	Not available
142	1.6	0.92	No	No	No	Not available	0
143	60.0	66.07	No	No	No	Not available	Not available
144	7.8	1.82	No	No	Yes	30	Not available
145	27.3	1.69	No	No	Yes	Not available	Not available
146	6.2	1.00	BRCA2 p.(Pro2767HisfsTer11)	No	No	Not available	Not available
147	9.4	0.90	No	No	No	Not available	10
148	6.2	4.63	No	No	Yes	Not available	Not available
149	4.7	4.35	No	No	No	Not available	Not available
150	1.6	1.87	No	CHEK2 p.(Lys373Glu)	No	Not available	Not available
151	4.7	1.89	No	No	Yes	Not available	Not available
152	2.3	1.89	No	No	No	Not available	Not available
153	2.4	3.70	No	No	No	0	Not available
154	4.7	1.75	No	No	No	1	Not available
155	12.5	0.92	No	No	Yes	3	Not available
156	7.1	0.90	No	No	No	80	Not available
157	5.5	0.89	No	ATM p.(Arg337Cys)	No	2	Not available
158	5.6	5.97	No	No	Yes	40	Not available
159	0.0	0.00	No	No	No	60	Not available
160	8.0	1.54	No	No	No	8	Not available
161	1.6	0.00	No	No	No	0	Not available
162	3.1	5.61	No	No	Yes	15	Not available

Note: Gene variants are described using HGVS-style protein nomenclature where applicable; splice-site variants are described using cDNA nomenclature. Gene fusions are uniformly denoted as GENE1::GENE2 fusion. TMB-High, MSI-High, and GIS-High status entries were not repeated in the Targetable alteration column when already captured in separate biomarker columns. Sampling sites and diagnostic terms were standardized by removing underscores, inconsistent capitalization, and extraneous spaces. TMB values are shown to one decimal place, and MSI values are shown to two decimal places.

Supplementary Table S3. Additional statistical analyses for GIS score associations

Table S3A. Frequency of GIS-High tumors with exact binomial 95% confidence intervals.

Group	Total cases, n	GIS-High cases, n	Frequency, %	Exact 95% CI, %
Overall cohort	162	14	8.6	4.8-14.1
Ovarian cancer	17	7	41.2	18.4-67.1
Non-ovarian tumors	145	7	4.8	2.0-9.7

Table S3B-1. Biomarker association analyses for GIS score in the overall cohort.

Biomarker	Group	n	Median GIS	Q1	Q3	Raw P value	BH-adjusted P value	Effect size r	Magnitude
BRCA1/2 alteration	No	154	14	5.0	23.8	3.38e-05	5.07e-05	0.326	Moderate
BRCA1/2 alteration	Yes	8	56	43.8	58.2				
TP53 mutation	No	81	8	3.0	14.0	1.91e-10	5.72e-10	0.501	Large
TP53 mutation	Yes	81	22	15.0	30.0				
Non-BRCA HRD-related gene alteration	No	155	14	5.5	26.0	0.595	0.595	0.042	Small
Non-BRCA HRD-related gene alteration	Yes	7	14	11.5	15.0				

BH, Benjamini-Hochberg; HRD, homologous recombination deficiency; Q1/Q3, first/third quartile. P values were calculated using the Wilcoxon rank-sum test.

Table S3B-2. Spearman correlation analyses in the overall cohort.

Comparison	Spearman rho	Raw P value	BH-adjusted P value
GIS score vs. TMB score	0.231	0.00311	0.00622
GIS score vs. MSI score	-0.030	0.704	0.704

Table S3C-1. Sensitivity analysis excluding ovarian cancers: biomarker group comparisons.

Biomarker	Group	n	Median GIS	Q1	Q3	Raw P value	BH-adjusted P value
BRCA1/2 alteration	No	139	13	5.0	22.0	0.000176	0.000264
BRCA1/2 alteration	Yes	6	53	33.2	57.0		
TP53 mutation	No	76	9	2.75	14.0	1.84e-07	5.53e-07
TP53 mutation	Yes	69	20	11.0	27.0		
Non-BRCA HRD-related gene alteration	No	138	14	5.0	23.8	0.740	0.740
Non-BRCA HRD-related gene alteration	Yes	7	14	11.5	15.0		

P values were calculated using the Wilcoxon rank-sum test.

Table S3C-2. Sensitivity analysis excluding ovarian cancers: Spearman correlation analyses.

Comparison	Spearman rho	Raw P value	BH-adjusted P value
GIS score vs. TMB score	0.237	0.00416	0.00832
GIS score vs. MSI score	-0.051	0.539	0.539

Table S3D. Specimen type analysis for GIS score.

Analysis cohort	Specimen group	n	Median GIS	Q1	Q3	Wilcoxon P value
Overall cohort	Resection	66	10.5	4.25	24.0	0.0853
Overall cohort	Biopsy/Cytology	96	17.0	10.0	26.0	
Non-ovarian tumors	Resection	51	8.0	3.5	20.0	0.00450
Non-ovarian tumors	Biopsy/Cytology	94	17.0	10.0	25.8	

Because only five cytology specimens were included, biopsy and cytology specimens were combined as a small-specimen group for this analysis.

Table S3E-1. Multivariable linear regression for GIS score in the overall cohort.

Variable	Estimate beta	Standard error	95% CI	P value
Intercept	-0.75	5.65	-11.9 to 10.4	0.894
Specimen group: Biopsy/Cytology vs. Resection	0.21	2.34	-4.41 to 4.83	0.929
BRCA1/2 alteration: Yes vs. No	25.10	4.50	16.2 to 34.0	1.09e-07
TP53 mutation: Yes vs. No	10.70	2.07	6.61 to 14.8	7.34e-07
Tumor group: Ovary vs. Others	17.60	4.15	9.44 to 25.9	3.74e-05
Tumor group: Breast vs. Others	12.10	3.81	4.59 to 19.6	0.00178
Tumor group: Lung vs. Others	0.11	2.65	-5.12 to 5.34	0.966
Tumor group: Hepatobiliary vs. Others	-0.94	3.85	-8.55 to 6.67	0.808
Tumor group: Uterus vs. Others	-5.95	3.99	-13.8 to 1.94	0.139
Age, per year	0.15	0.08	-0.01 to 0.31	0.0728
Sex: Male vs. Female	0.94	2.50	-3.99 to 5.88	0.707

Model summary: adjusted R-squared = 0.469. Reference categories: specimen group, resection; BRCA1/2 alteration, No; TP53 mutation, No; tumor group, Others; sex, Female.

Table S3E-2. Sensitivity analysis excluding ovarian cancers: multivariable linear regression for GIS score.

Variable	Estimate beta	Standard error	95% CI	P value
Intercept	2.76	4.98	-7.09 to 12.6	0.580
Specimen group: Biopsy/Cytology vs. Resection	0.55	2.08	-3.56 to 4.67	0.791
BRCA1/2 alteration: Yes vs. No	27.10	4.42	18.3 to 35.8	9.40e-09
TP53 mutation: Yes vs. No	7.90	1.86	4.21 to 11.6	4.12e-05
Tumor group: Breast vs. Others	11.70	3.29	5.17 to 18.2	0.000532
Tumor group: Lung vs. Others	0.82	2.29	-3.70 to 5.35	0.719
Tumor group: Hepatobiliary vs. Others	0.03	3.33	-6.55 to 6.61	0.992
Tumor group: Uterus vs. Others	-6.11	3.45	-12.9 to 0.71	0.0787
Age, per year	0.10	0.07	-0.04 to 0.25	0.162
Sex: Male vs. Female	1.22	2.16	-3.04 to 5.48	0.572

Model summary: adjusted R-squared = 0.414. Reference categories: specimen group, resection; BRCA1/2 alteration, No; TP53 mutation, No; tumor group, Others; sex, Female.

Statistical notes: Frequencies were calculated with exact binomial 95% confidence intervals. Group comparisons were performed using the Wilcoxon rank-sum test. Spearman rho was used as an effect-size estimate for correlation analyses. Benjamini-Hochberg adjustment was applied to exploratory biomarker association analyses. Multivariable linear regression models included specimen group, BRCA1/2 alteration, TP53 mutation, tumor group, age, and sex.
